# ZFP36 Protects against Abdominal Aortic Aneurysm Formation by Regulating Vascular Smooth Muscle Phenotypic Switch

**DOI:** 10.34133/research.1078

**Published:** 2026-01-16

**Authors:** Zhinan Wu, Hanlin Lu, Tingting Liu, Xiaolin Yue, Lei Wang, Chang Ma, Pavel Kovarik, Xiangjiu Ding, Mo Wang, Cheng Zhang, Jianmin Yang, Wencheng Zhang

**Affiliations:** ^1^ State Key Laboratory for Innovation and Transformation of Luobing Theory; Key Laboratory of Cardiovascular Remodeling and Function Research of MOE, NHC, CAMS and Shandong Province; Department of Cardiology, Qilu Hospital of Shandong University, Jinan 250012, China.; ^2^ Max Perutz Labs, University of Vienna, Vienna Biocenter (VBC), A-1030 Vienna, Austria.; ^3^ Department of Vascular Surgery, General Surgery, Qilu Hospital of Shandong University, Jinan 250012, China.; ^4^ Key Laboratory of Rheumatic Immune Disease Medical Prevention Integration and Translation of Shandong Province, Qilu Hospital of Shandong University, Jinan 250012, China.; ^5^ Department of Vascular Surgery, Shandong Provincial Hospital Affiliated to Shandong First Medical University, Jinan 250021, China.

## Abstract

Abdominal aortic aneurysm (AAA) is a life-threatening aortic disease without effective pharmacological therapies. Mounting evidences suggested that RNA-binding proteins (RBPs) exert pivotal roles in various diseases including AAA. The public human AAA microarray dataset and the RBP database were used to screen the involved RBPs during AAA formation. The integrated analysis identified zinc finger protein 36 (ZFP36) as a potential mediator of AAA. Underexpression of ZFP36 was observed in aortic vascular smooth muscle cells (VSMCs) within aneurysms from patients and angiotensin II (AngII)-induced mice. *Zfp36* deficiency in VSMCs augmented extracellular matrix (ECM) degeneration, VSMC phenotypic switch, and apoptosis, which promoted AAA formation in the AngII-infused model. In contrast, VSMC-specific overexpressing *Zfp36* inhibited AAA formation. Mechanically, guanylate binding protein 2 (GBP2), a GTPase related to interferon-γ signaling, was identified as a direct target gene of ZFP36 by analyzing the bulk sequencing data. We confirmed that ZFP36 regulates VSMC phenotypic switch via manipulating Yes-associated protein1/TEA domain transcription factor 1 (YAP1/TEAD1) signaling in a GBP2-dependent manner. Additionally, we further verified that dexamethasone (Dex) could promote glucocorticoid receptor nuclear translocation and *Zfp36* transcription. In vivo Dex administration prevented AAA formation in a ZFP36-dependent manner. These findings revealed the regulatory role of ZFP36/GBP2/YAP1/TEAD1 signaling in VSMC phenotypic switch and AAA formation, and provided a novel strategy (Dex) for AAA treatment.

## Introduction

Abdominal aortic aneurysm (AAA) is characterized by a focal weakening and expansion of the abdominal aorta [1]. Most AAA has no symptoms, but its mortality exceeds 80% once rupture [2]. To date, AAA has become a major cause of disability and death worldwide and its incidence is still climbing. The clinical intervention of AAA is limited to surgery including open or endovascular repair, and the effective pharmacological strategies are still lacking, which emphasized the urgency of identifying novel targets for AAA treatment.

Vascular smooth muscle cells (VSMCs), the main component of tunica media, plays an important role in maintaining vascular homeostasis [3]. It is well known that VSMC is a differentiated cell type with remarkable plasticity. In healthy vessel wall, VSMCs exhibit a contractile phenotype characterized by high expression of contractile proteins (alpha-smooth muscle actin [α-SMA], smooth muscle 22 alpha [SM22α], and myosin heavy chain-11 [MYH11]) [4,5]. Upon injury, VSMCs switch to a synthetic phenotype. This kind of VSMCs could release matrix metalloproteinases (MMPs) and proinflammatory cytokines, which cause inflammatory cell migration and extracellular matrix (ECM) degradation [6]. The VSMC phenotypic switch is fundamental to the pathogenesis of many aortic diseases such as AAA; therefore, understanding its regulatory mechanisms is necessary for developing therapeutic strategies in AAA treatment.

RBPs (RNA-binding proteins) are critical effectors of gene expression, which could manipulate all aspects of RNA, including transcription, splicing, modification, intracellular trafficking, translation, and decay [7,8]. It has been proven that the malfunction of RBPs initiated various diseases including AAA, and it could be promising targets for therapeutic strategy development [9]. Zinc finger protein 36 (ZFP36), is one of the RBPs that belong to the AU-rich element RNA-binding protein (AUBP) family, which bind to the preferred AU-rich element within the 3′-untranslated regions (UTRs) to promote mRNA deadenylation and decay [10]. ZFP36 has gathered widespread attention for its superb anti-inflammatory effects in many autoimmune diseases [11]. The protective roles of ZFP36 in inhibiting foam cell formation and atherosclerosis have also been reported [12,13]. However, the involvement between ZFP36 and VSMC phenotypic switch and its participation in AAA formation remains elusive.

In the present study, we found that ZFP36 could manipulate VSMC phenotypic switch and inhibit AAA formation. Guanylate binding protein 2 (GBP2) was identified as the downstream factor of ZFP36 by bulk sequencing and RNA immunoprecipitation, which disturbed the YAP1 (yes-associated protein 1)/TEAD1 (TEA domain transcription factor 1) signaling and promoted VSMC dysfunction and AAA progression. Additionally, we found that dexamethasone (Dex) could be an effective drug in treating AAA in a ZFP36-dependent manner. Taken together, our study provides fundamental insights into the underlying mechanisms in AAA formation and offered a novel strategy for AAA treatment.

## Results

### ZFP36 is down-regulated during AAA progression

**Fig. 1.**
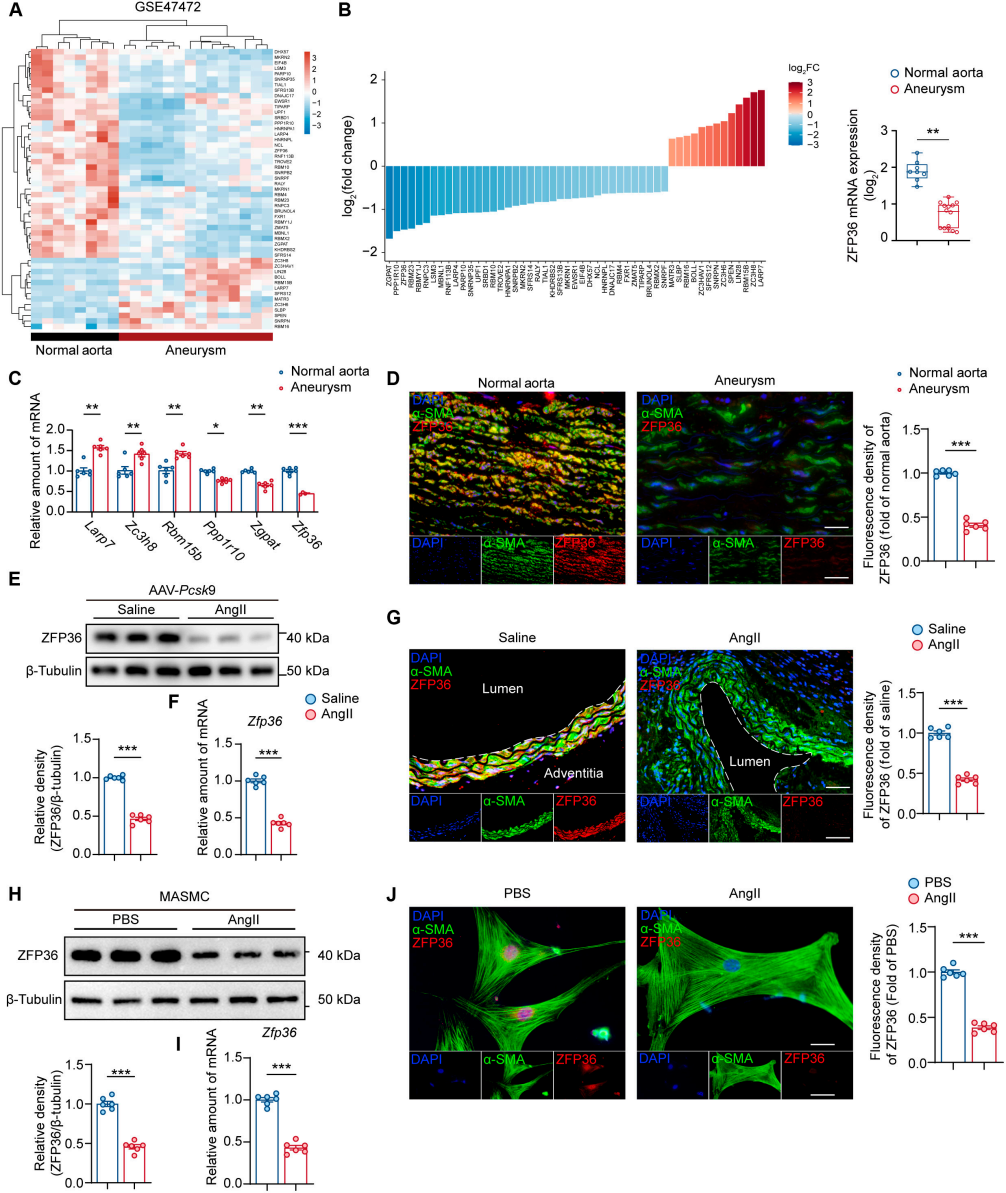
ZFP36 is down-regulated during AAA progression. (A and B) Relative mRNA expression of RNA-binding proteins among DEGs in the microarray dataset (GSE47472). The data were log2-transformed. The log2 FC and *P* values were calculated, and multiple testing using the Benjamini–Hochberg method was applied to adjust the *P* values. With a Benjamini–Hochberg-adjusted *P* < 0.05 and |log2FC| ≥ 1 as the criteria. (C) RT-qPCR validation of the top 3 up-regulated genes and the top 3 down-regulated genes in (B) between murine normal aorta and AAA (*n* = 6 per group). Statistical comparisons were performed using unpaired *t* tests. The false discovery rate (FDR) was controlled for multiple comparisons using the 2-stage step-up method of Benjamini, Krieger, and Yekutieli. (D) Representative images of ZFP36 expression by IF staining of human AAA and normal aorta sections and quantification of fluorescence intensity. Scale bar indicates 50 μm (*n* = 6 per group). (E) Protein levels of ZFP36 in murine normal aorta and AAA (*n* = 6 per group). (F) Relative mRNA level of *Zfp36* in murine normal aorta and AAA (*n* = 6 per group). (G) Representative images of ZFP36 expression by IF staining of mouse AAA and normal aorta sections and quantification of fluorescence intensity. Scale bar indicates 50 μm (*n* = 6 per group). (H and I) Protein levels and relative mRNA level of *Zfp36*of ZFP36 in VSMCs treated with PBS or AngII (1 μM) for 48 h (*n* = 6 per group). (J) Representative images of ZFP36 expression by IF staining of mouse VSMCs treated with PBS or AngII (1 μM) for 48 h and quantification of fluorescence intensity (*n* = 6 per group). Scale bar indicates 20 μm. Statistical analyses of (D) to (J) were performed using unpaired *t* test. ns indicates not significant; *P < 0.05; **P < 0.01; ***P < 0.001.

To identify candidate RBPs involved in AAA progression, we performed integrated analysis using the microarray dataset GSE47472 and the human RBP dataset (http://rbpmap.technion.ac.il/) [14]. Differentially expressed genes (DEGs) of RBPs were visualized by a heatmap and a bar chart (Fig. 1A and B). Thus, we selected the top 3 up-regulated genes including *Larp7*, *Zc3h8*, and *Rbm15b* and the top 3 down-regulated genes including *Ppp1r10*, *Zgpat*, and *Zfp36* as the candidate genes to verify in a murine AAA model. Real-time quantitative PCR (RT-qPCR) results showed that the expression alteration of *Zfp36* between normal aorta and aneurysm is most significant among candidates (Fig. 1C). Immunofluorescence (IF) staining revealed that ZFP36 was primarily expressed in VSMCs within the vasculature and was markedly down-regulated in aneurysm (Fig. 1D). We further confirmed the expression profile of ZFP36 in the angiotensin II (AngII)-induced AAA model, and the results of Western blotting (WB), RT-qPCR, and IF were consistent with former findings, indicating a remarkable decreased expression of ZFP36 in aneurysm (Fig. 1E to G). In vitro experiments also confirmed the down-regulation of ZFP36 in VSMCs after AngII stimulation (Fig. 1H to J).

Taken together, we found that *Zfp36* is highly expressed in aortic VMSCs, and ZFP36 is significantly down-regulated during AAA progression or AngII stimulation, which suggested its involvement in VSMC dysfunction and AAA formation.

### ZFP36 in VSMCs prevented AAA progression

**Fig. 2.**
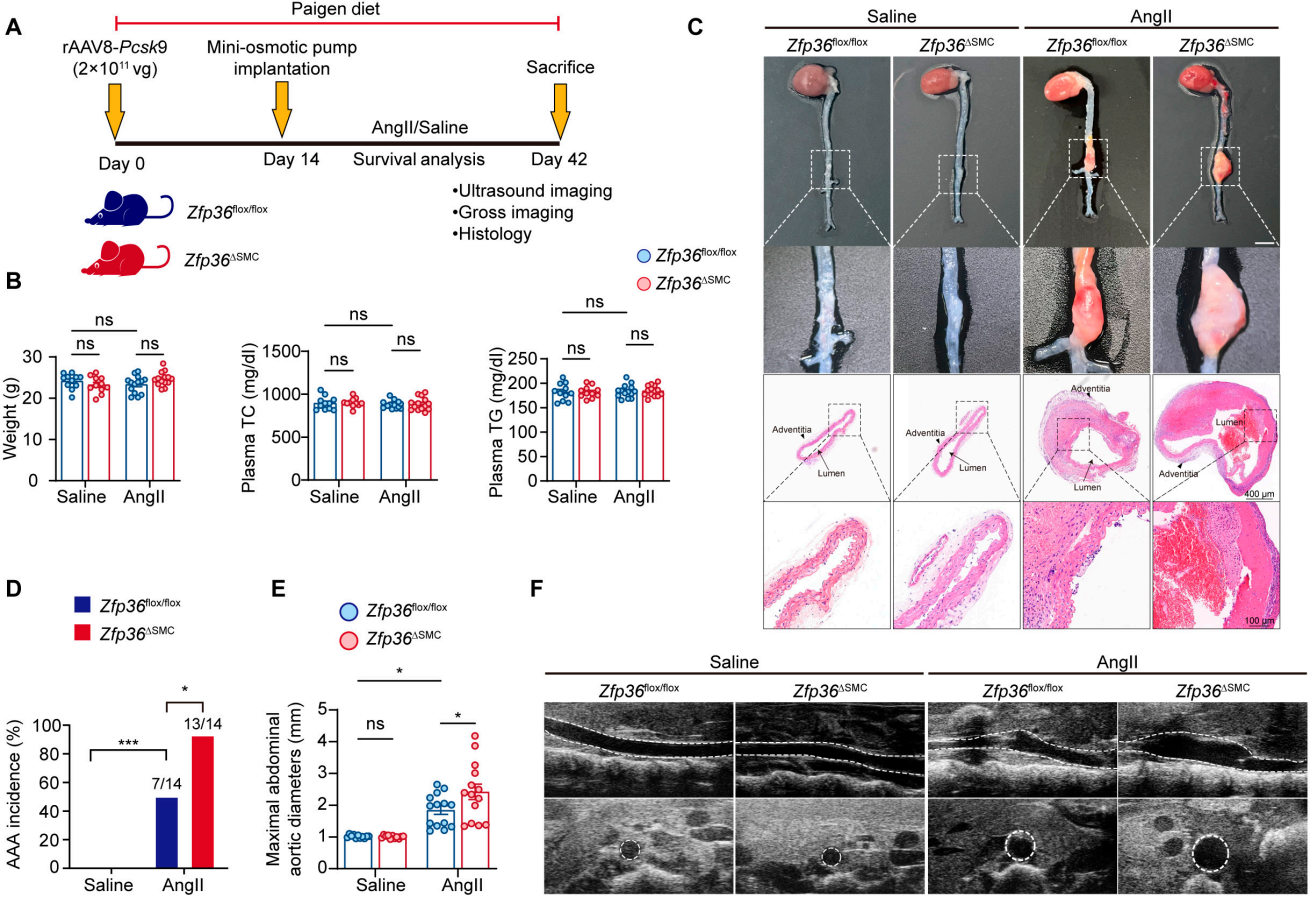
VSMC-specific *Zfp36* deletion augmented AngII-induced AAA formation. (A) Diagram of the experimental procedure. (B) Body weights, total cholesterol, and total triglyceride of plasma in *Zfp36*^△SMC^ mice and *Zfp36*^flox/flox^ mice treated with saline or AngII (*n* = 11 to 14 per group). Statistical analyses were analyzed by 2-way analysis of variance (ANOVA) following Tukey’s multiple comparisons. (C) Representative images of macroscopic features and HE staining of cross-sections of abdominal aortas. Scale bar indicates 4 mm. (D) Incidence of AAA induced by AngII in indicated groups (*n* = 11 for saline administration; *n* = 14 for AngII administration). Data were analyzed by a Fisher exact test. (E) Quantification of the maximal diameter of suprarenal abdominal aortas (*n* = 11 for saline administration; *n* = 14 for AngII administration). Data were analyzed by 2-way ANOVA following Tukey’s multiple comparisons test. (F) Representative images of abdominal aortas visualized by using the ultrasound imaging in indicated groups. ns indicates not significant; *P < 0.05; ***P < 0.001.

To examine the potential role of ZFP36 in AAA progression, we constructed VSMC-specific *Zfp36*-deficient mice (*Zfp36*^△SMC^) and the littermate control mice (*Zfp36*^flox/flox^) (Fig. S1A to G) and established an AAA model following a previous reported protocol [15]. Briefly, male *Zfp36*^△SMC^ mice and *Zfp36*^flox/flox^ mice were previously injected with rAAV8/D377Y-mPCSK9 and fed Paigen diet for 2 weeks to induce hypercholesterolemia. Then, *Zfp36*^△SMC^ mice and *Zfp36*^flox/flox^ mice were randomly subdivided into 2 groups and implanted osmosis pump infused with saline or AngII (1,000 ng/kg/min; 28 days) (Fig. 2A). As shown in Fig. 2B, no differences in body weight, serum cholesterol, and serum triglycerides were observed between *Zfp36*^△SMC^ mice and *Zfp36*^flox/flox^ mice. Of interest, VSMC-specific *Zfp36* deficiency led to a significant increase in AAA incidence, and the mean maximal abdominal aortic diameter (Fig. 2C to F), which suggested that ZFP36 in VSMCs protected against AAA formation. An alternative model of AAA induced by PPE (porcine pancreatic elastase) was used to comprehensively validate the effects of VSMC-specific *Zfp36* deletion in AAA development (Fig. S2A). In accordance with previous results, *Zfp36*^△SMC^ mice displayed augmented aneurysmal lesion compared to *Zfp36*^flox/flox^ mice after PPE induction (Fig. S2B to D).

To further determine the beneficial effects of ZFP36 in VSMCs, we constructed AAV2-Ctrl and AAV2-*Zfp36* under the *Tagln* promoter. As shown in Fig. S3A, male mice were randomly subdivided into 2 groups and injected with AAV2-Ctrl or AAV2-*Zfp36.* Subsequently, the AAA model was established as previously described. At the end of the induction period, we recorded significant reductions in the mean maximal abdominal aortic diameter in the AAV2-*Zfp36* group (Fig. S3B to D). Taken together, our results strongly suggested that VSMC-derived *Zfp36* acts as an endogenous benign factor that protected against the pathogenesis of AAA.

### VSMC-specific *Zfp36* augmented AngII-induced aortic remodeling

**Fig. 3.**
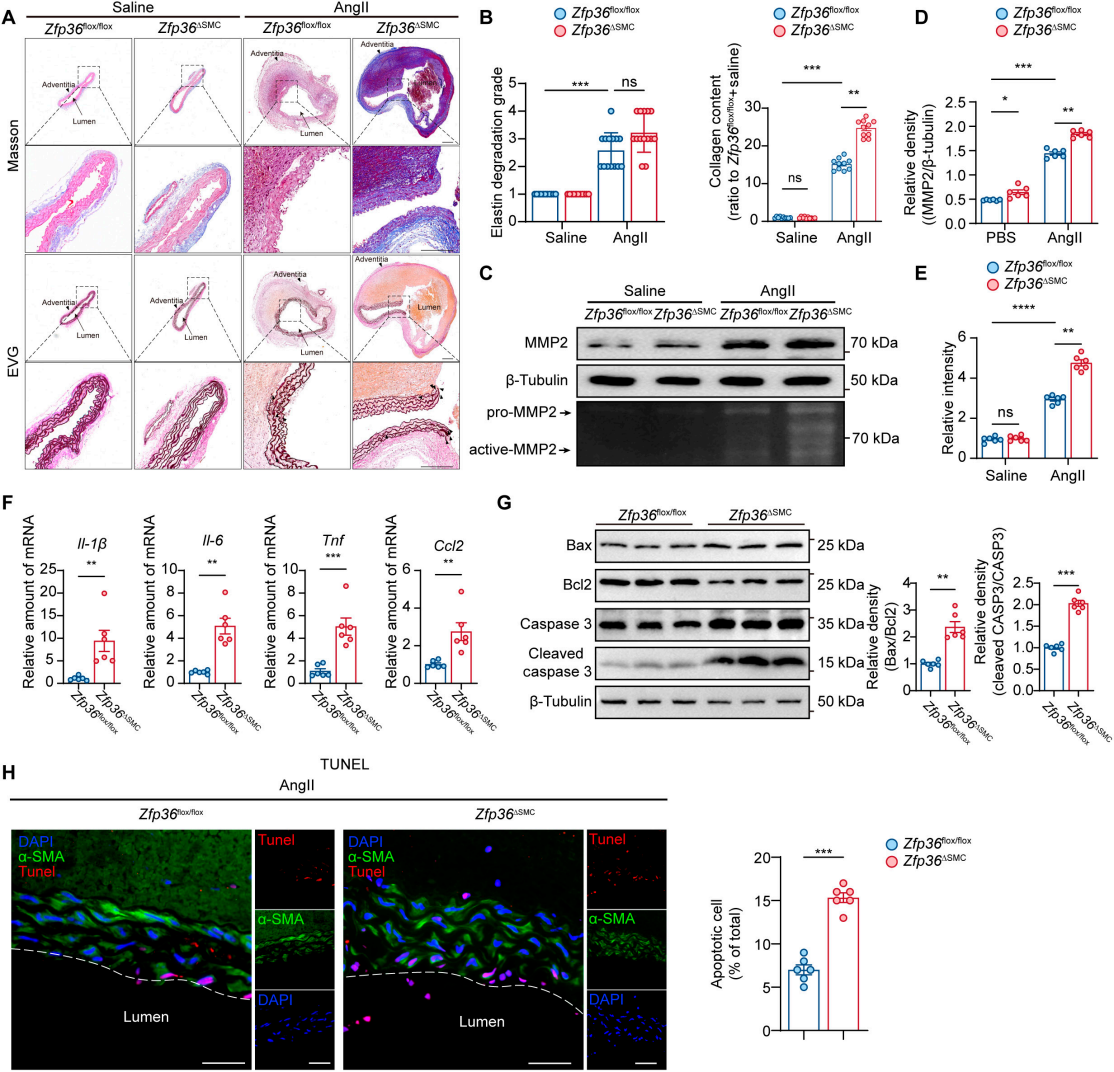
VSMC-specific *Zfp36* deletion augmented AngII-induced aortic remodeling. (A) Representative images of Masson and EVG staining of suprarenal abdominal aortas of mice in indicated groups. Scale bar indicates 100 μm. (B) Grade of elastin degradation (*n* = 10 per group). Data were analyzed by nonparametric Kruskal–Wallis test with Dunn’s post-hoc test, and quantitative analysis of collagen deposition (*n* = 10 per group). Data were expressed as the mean ± SEM and analyzed by 2-way ANOVA following Tukey’s multiple comparisons test. (C) Protein levels of MMP2 of abdominal aortic tissues in indicated groups (*n* = 6 per group) and activities of MMPs of abdominal aortic tissues in indicated groups were determined by a gelatin zymography assay (*n* = 6 per group). (D and E) Quantification of MMP2 protein level and activities in (C). Data analyses of (D) and (E) are performed using 2-way ANOVA following Tukey’s multiple comparisons test. (F) Relative mRNA level of *Il-1β*, *Il-6*, *Tnf*, and *Ccl-2* of abdominal aortic tissues in indicated groups (*n* = 6 per group). (G) Protein levels of Bax, Bcl2, Caspase 3, and cleaved Caspase 3 of abdominal aortic tissues in indicated groups (*n* = 6 per group). (H) TUNEL staining of suprarenal abdominal aortas of mice in indicated groups (*n* = 6 per group). Scale bar indicates 50 μm. Statistical analyses of (F) and (G) were performed using unpaired *t* test. ns indicates not significant; *P < 0.05; **P < 0.01; ***P < 0.001; ****P < 0.0001.

ECM degradation, inflammation, and VSMC apoptosis are the fundamentals of AAA pathogenesis [16]. We performed Masson staining and elastic Van Gieson (EVG) staining to evaluate the aortic remodeling degree; the results showed a significant increase of elastin degradation and collagen content in aorta of the *Zfp36*^△SMC^ group (Fig. 3A and B). MMPs are the main contributors of aortic remodeling [17], and the main subtypes within the aortic wall are MMP2 and MMP9. It is well known that VSMC is the main origin of MMP2 while macrophage produces more MMP9. Thus, we determined the effect of ZFP36 deficiency on MMP2; WB results showed that VSMC-specific *Zfp36* knockout augmented MMP2 up-regulation induced by AngII (Fig. 3C and D). Besides, MMP activity was determined by gelatin zymography assay, which also indicated higher MMP activity in the *Zfp36*^△SMC^ group (Fig. 3C and E). Considering *Zfp36* is a well-known anti-inflammatory gene, we examined the inflammatory factors related to AAA progression. RT-qPCR results showed that the expression levels of *Il-1β*, *Il-6*, *Tnf*, and *Ccl-2* in aortic tissues from the *Zfp36*^△SMC^ group were much higher (Fig. 3F). Loss of VSMCs in media layer led to the aortic wall weakening and eventually aneurysm rupture [18]. WB results showed increased Bax/Bcl2 ratio and cleaved Caspase 3/total Caspase 3 ratio, which indicated that ZFP36 deficiency promoted cell apoptosis with aortic tissue (Fig. 3G). TdT-mediated dUTP nick-end labeling (TUNEL) assay indicated more apoptotic VSMCs in aortic wall from *Zfp36*^△SMC^ mice (Fig. 3H), which further suggested that ZFP36 deficiency accelerated VSMC apoptosis and loss.

Severe elastin degradation and collagen deposition were observed in *Zfp36*^△SMC^ mice of the PPE model (Fig. S3D to F). Besides, we also confirmed the effects of ZFP36 overexpressing on aortic remodeling. Mice injected with AAV2-*Zfp36* showed less elastin degradation and milder collagen deposition in aortic tissues (Fig. S3E to G). Taken together, our results demonstrated that ZFP36 could inhibit AngII-induced aortic remodeling via suppressing ECM degradation, inflammation, and VSMC apoptosis.

### ZFP36 manipulated VSMC phenotypic switch

**Fig. 4.**
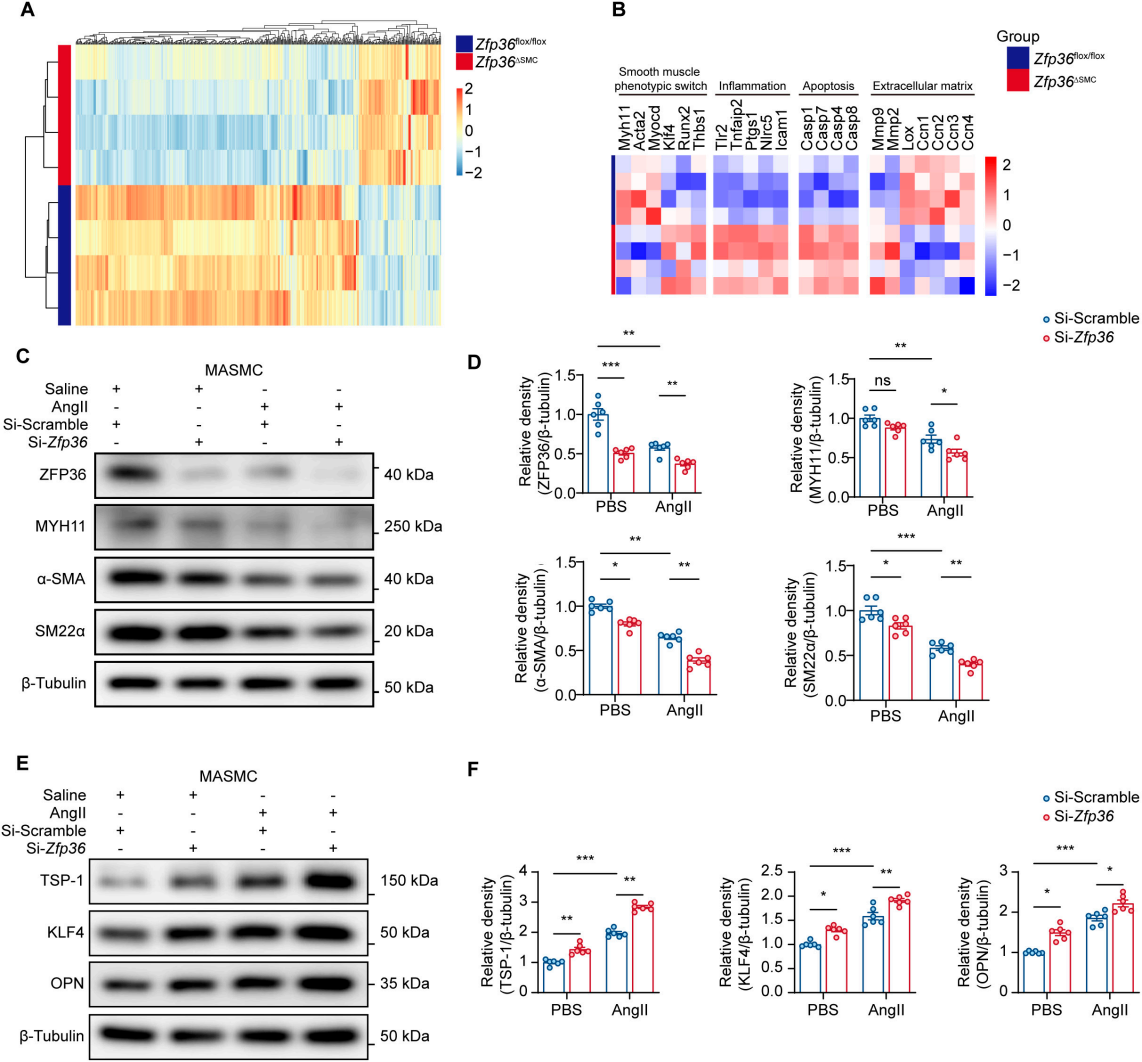
ZFP36 knockdown accelerated AngII-induced VSMC phenotypic switch. (A) Heatmap of DEGs in the *Zfp36*^△SMC^ group and the *Zfp36*^flox/flox^ group (*n* = 4 per group). (B) Visualization of DEGs related to smooth muscle phenotypic switch, inflammation, apoptosis, and extracellular matrix. (C and D) Expressions of contractile phenotype-related proteins of VSMCs transfected with Si-Scramble or Si-*Zfp36* and treated with saline or AngII (1 μM) for 48 h (*n* = 6 per group), and quantification of protein expression levels was analyzed by 2-way ANOVA following Tukey’s multiple comparisons test. (E and F) Expressions of synthetic phenotype-related proteins of VSMCs transfected with Si-Scramble or Si-*Zfp36* and treated with saline or AngII (1 μM) for 48 h (*n* = 6 per group), and quantification of protein expression levels was analyzed by 2-way ANOVA following Tukey’s multiple comparisons test. ns indicates not significant; *P < 0.05; **P < 0.01; ***P < 0.001.

To unveil the underlying mechanisms of ZFP36 protecting against AAA, we performed bulk sequencing using aortas from *Zfp36*^△SMC^ mice and *Zfp36*^flox/flox^ mice (Fig. 4A). As expected, genes related to inflammation (*Tlr2*, *Tnfaip2*, *Ptgs1*, *Nlrc5*, and *Icam1*), ECM degradation (*Mmp2* and *Mmp9*), and apoptosis (*Casp1*, *Casp4*, *Casp7*, and *Casp8*) showed great up-regulation in aortic tissues from *Zfp36*^△SMC^ mice, while genes related to ECM synthesis (*Lox*, *Ccn1*, *Ccn2*, *Ccn3*, and *Ccn4*) were down-regulated (Fig. 4B). Of note, genes related to the VSMC phenotype showed remarkable change between 2 groups. Contractile phenotype-related genes including *Myh11*, *Acta2*, and *Myocd* showed lower expression levels in aortas from *Zfp36*^△SMC^ mice, while synthetic phenotype-related genes including *Klf4*, *Runx2*, and *Thbs1* all up-regulated. The above results suggested that ZFP36 may be a regulator of VSMC phenotypic switch. We transfected siRNA of *Zfp36* into primary mouse aortic VSMCs and subsequently administrated AngII. WB results showed that knockdown of *Zfp36* augmented down-regulation of MYH11, α-SMA, and SM22α induced by AngII (Fig. 4C and D). Thrombospondin-1 (TSP-1), Krüppel-like factor 4 (KLF4), and osteopontin (OPN) showed remarkable increase after *Zfp36* knockdown (Fig. 4E and F).

To verify the regulatory role of ZFP36 on VSMC phenotypic switch, we further constructed control adenovirus Ad-Ctrl and overexpressing adenovirus Ad-*Zfp36.* WB results were consistent with our previous conclusion, that is, overexpression of *Zfp36* up-regulated contractile phenotypic proteins (MYH11, α-SMA, and SM22α) (Fig. S4A and B) and down-regulated synthetic phenotypic proteins (TSP-1, KLF4, and OPN) (Fig. S4C and D), inhibiting AngII-induced VSMC phenotypic switch. Taken together, our results demonstrated that ZFP36 is a key regulator of VSMC phenotypic switch.

### Identification of GBP2 as a target of ZFP36 during AAA progression

**Fig. 5.**
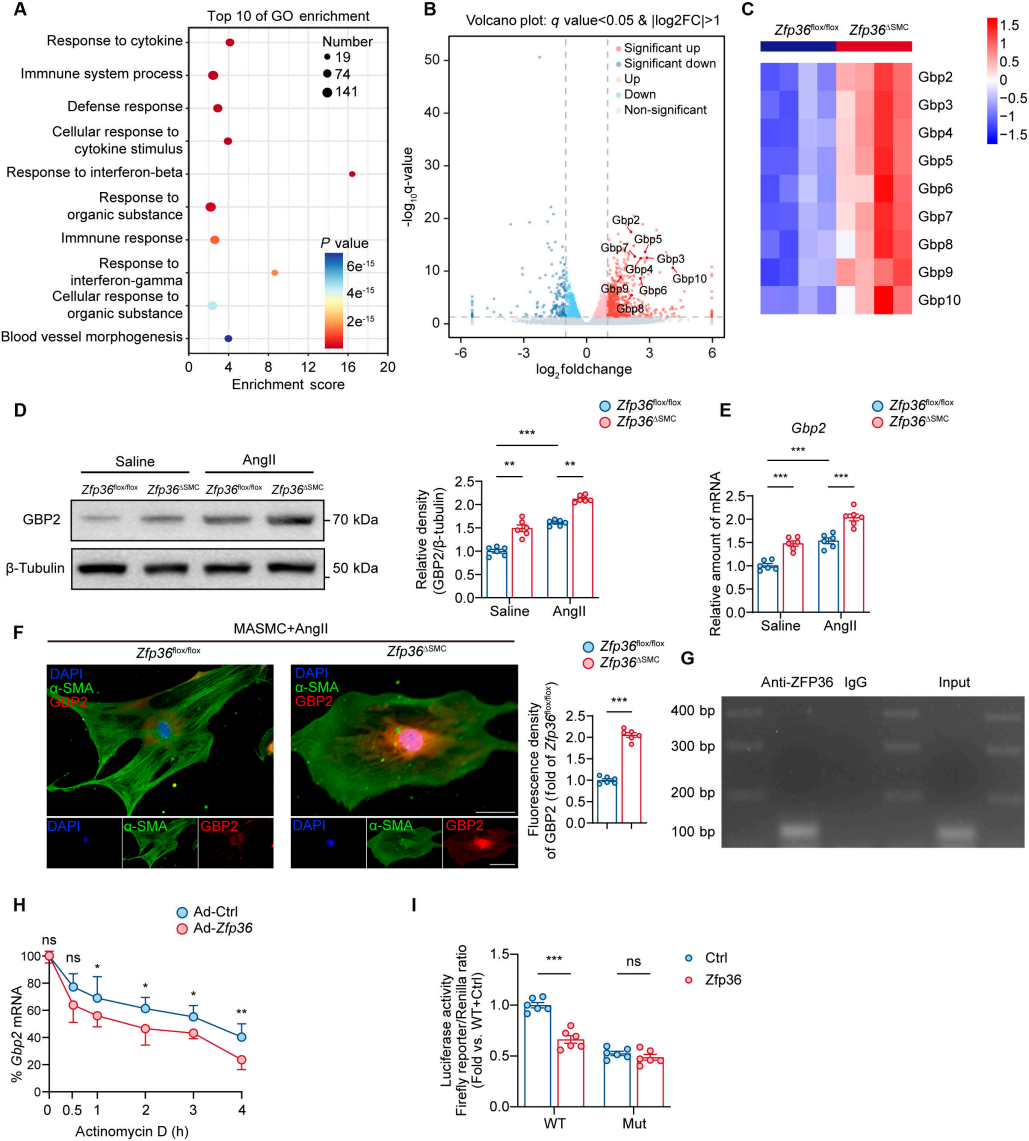
GBP2 is the direct target of ZFP36 during AAA progression. (A) The top 10 Gene Ontology (GO) enrichment pathways of differentially expressed genes. (B) Volcano plot reveals altered genes in bulk sequencing. Differentially expressed genes were defined as genes with a Benjamini–Hochberg-adjusted *P* value <0.05 and |log2(FC)| ≥ 1. (C) Visualization of DEGs of the GBP family. (D) Expression of GBP2 in aortic tissues (*n* = 6 per group). (E) Relative mRNA expression of *Gbp2* in aortic tissues (*n* = 6 per group). Analyses of protein expression in (D), and mRNA expression in (E) were performed by 2-way ANOVA following Tukey’s multiple comparisons test. (F) Representative images of GBP2 expression by IF staining of VSMCs and quantification (*n* = 6 per group). Scale bar indicates 20 μm. Statistical analysis was performed by unpaired *t* test. (G) RNA immunoprecipitation was performed with antibodies to ZFP36, and the target 3′UTR region of *Gbp2* was amplified by PCR. (H) Relative mRNA expression of *Gbp2* in indicated groups infected with Ad-Ctrl or Ad-*Zfp36* for 48 h and treated with actinomycin D (ActD, 10 μg/ml) for different periods. Statistical analysis was performed by unpaired *t* test. (I) Luciferase reporter assay. Firefly luciferase activity was normalized to Renilla activity and expressed as relative luciferase activity (*n* = 6 per group). Statistical analysis was performed by 2-way ANOVA following Tukey’s multiple comparisons test. ns indicates not significant; *P < 0.05; **P < 0.01; ***P < 0.001.

To delve into the underlying mechanisms of ZFP36 mediating VSMC phenotypic switch, we further analyzed the bulk sequencing data; Gene Ontology (GO) analysis showed that most multiple immune and inflammation pathways changed (Fig. 5A). Among the DEGs, we noticed the guanylate binding protein (GBP) family; most members of this family (*Gbp2* to *Gbp10*) showed great up-regulation in aortas from *Zfp36*^△SMC^ mice (Fig. 5B and C). Thus, we speculated that ZFP36 may exert its function via regulating GBPs. We examined the expression alteration of *Gbps* in vivo and in vitro after AngII administration. RT-qPCR results showed that *Gbp2* increased most significantly in aortic tissues from mice infused with AngII (Fig. S5A) or VSMCs treated with AngII (Fig. S5B). WB and IF further confirmed that GBP2 was up-regulated in AAAs especially VSMCs within the aortic wall (Fig. S5C and D). We verified the expression profile of GBP2 in *Zfp36*^△SMC^ mice and *Zfp36*^flox/flox^ mice infused with saline or AngII. WB and RT-qPCR both suggested that ZFP36 deficiency augmented GBP2 up-regulation induced by AngII (Fig. 5D and E). IF staining showed a significant stronger fluorescence intensity of GBP2 of VSMCs from *Zfp36*^△SMC^ mice compared to *Zfp36*^flox/flox^ mice (Fig. 5F).

The above results strongly suggested that GBP2 is the direct target of ZFP36. It is well-known that, as a member of AUBPs, ZFP36 binds to the 3′UTRs to promote mRNA decay. We performed RNA immunoprecipitation; the PCR results revealed that ZFP36 could bind to the 3′UTR of *Gbp2*’s mRNA (Fig. 5G). The results of the mRNA stability assay showed that *Gbp2*’s mRNA decays faster after *Zfp36* overexpression (Fig. 5H). Based on the prediction of the RNA-Binding Protein Database, the putative ZFP36 binding site was identified in *Gbp2* mRNA 3′UTR. To verify that ZFP36 directly binds to *Gbp2* mRNA 3′UTR, we performed dual luciferase reporter assay as previously described [19]. As expected, ZFP36 overexpression reduced the luciferase activity, whereas mutation of the 3′UTR diminished this effect (Fig. 5I). In vivo experiments were conducted to determine that the detrimental effects of ZFP36 deletion were mediated by GBP2. *Zfp36*^△SMC^ mice and *Zfp36*^flox/flox^ mice were injected with pAAV-SM22ap-MCS-mCherrymiR30shRNA (NC)-WPRE (AAV-ShNC) or pAAV-SM22ap-MCS-mCherrymiR30shRNA (Gbp2)-WPRE (AAV-Sh*Gbp2*) and subjected to AAA modeling (Fig. S6A). Knockdown of GBP2 in *Zfp36*^△SMC^ mice markedly ameliorated AAA progression caused by *Zfp36* deletion in VSMCs, and it also showed moderate effects in *Zfp36*^flox/flox^ mice (Fig. S6B and C). Histological analyses showed improved aortic remodeling in *Zfp36*^△SMC^ mice injected with AAV-Sh*Gbp2* compared to AAV-ShNC (Fig. S6D and E). Collectively, we identified GBP2 as the target of ZFP36, which is involved in AAA progression.

### ZFP36 inhibits VSMC phenotypic switch in a GBP2/YAP1/TEAD1-dependent manner

**Fig. 6.**
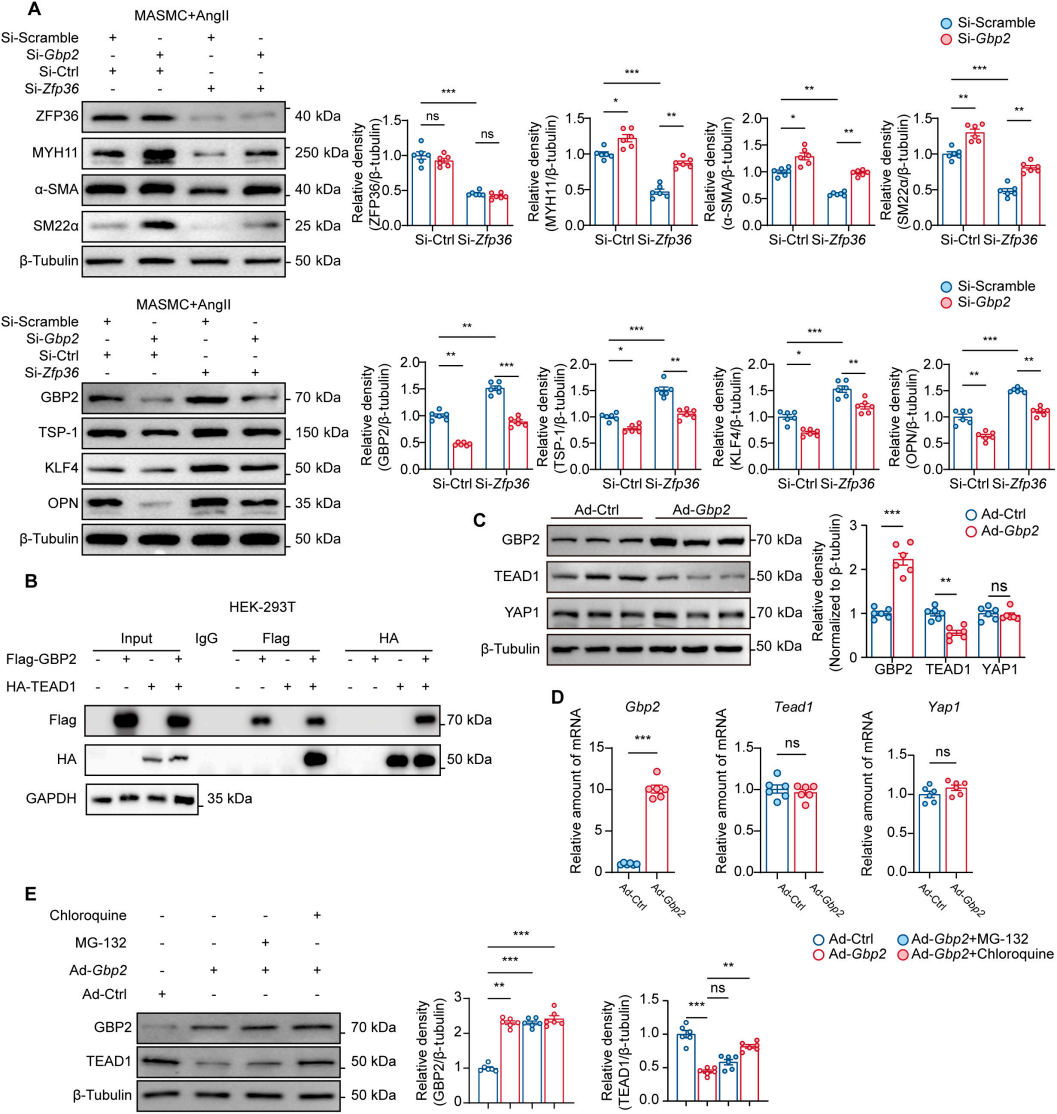
ZFP36 inhibits VSMC phenotypic switch in a GBP2/YAP1/TEAD1-dependent manner. (A) Expressions of contractile phenotype-related proteins and synthetic phenotype-related proteins in VSMCs treated with AngII (1 μM) for 48 h after transfection (*n* = 6 per group), and quantifications of protein expression levels were analyzed by 2-way ANOVA following Tukey’s multiple comparisons test. (B) Co-immunoprecipitation of HEK-293T cells transfected with Flag-GBP2 plasmid and HA-TEAD1 plasmid. (C) Protein levels of VSMCs transfecting with Ad-Ctrl or Ad-*Gbp2* (*n* = 6 per group)*.* (D) Relative mRNA levels in VSMCs transfecting with Ad-Ctrl or Ad-*Gbp2* (*n* = 6 per group)*.* Statistical analyses of (C) and (D) were performed by unpaired *t* test. (E) Protein levels of VSMCs infecting with Ad-Ctrl or Ad-*Gbp2* after MG-132 or chloroquine treatment (*n* = 6 per group). Statistical analysis was performed by 2-way ANOVA following Tukey’s multiple comparisons test. ns indicates not significant; *P < 0.05; **P < 0.01; ***P < 0.001.

To verify that ZFP36 regulates VSMC phenotypic switch in a GBP2-dependent manner, we transfected siRNA of *Zfp36* and *Gbp2* simultaneously into VSMCs and administrated AngII. WB results showed that *Gbp2* knockdown rescued the down-regulation of contractile proteins (MYH11, α-SMA, and SM22α) induced by *Zfp36* knockdown (top panel of Fig. 6A). Besides, the up-regulation of synthetic proteins (TSP-1, KLF4, and OPN) was almost reversed by *Gbp2* knockdown (bottom panel of Fig. 6A). These results further confirmed that ZFP36 manipulates VSMC phenotypic switch via regulating GBP2.

It is well-known that the regulatory network of VSMC phenotypic switch consists of multiple transcriptional factors [20], and the YAP1/TEAD1 complex is the most important among them [21]. A previous study has demonstrated that human GBP1, the homologue of mouse GBP2, could interact with TEADs [22]. Thus, we first examined the interaction between GBP2 and TEAD1 by transfecting Flag-GBP2 and HA-TEAD1 plasmid into HEK-293T cells. The results of coimmunoprecipitation showed the combination of GBP2 and TEAD1 (Fig. 6B). Of interest, the results of WB indicated that overexpression of *Gbp2* led to the down-regulation of TEAD1 without affecting YAP1 expression (Fig. 6C), while RT-qPCR results displayed no alteration of *Yap1* and *Tead1* after Gbp2 overexpression (Fig. 6D), which suggested that GBP2 could promote degradation of TEAD1. Considering GBP2 was reported to be involved in the ubiquitin–proteasome pathway and autophagy–lysosomal pathway, we overexpressed *Gbp2* and treated it with the proteasome inhibitor MG-132 or the autophagy inhibitor chloroquine (CQ); WB results showed that CQ restored TEAD1 expression after *Gbp2* overexpression (Fig. 6E), indicating that GBP2 may disturb the YAP1/TEAD1 complex by promoting TEAD1 degradation via the autophagy–lysosomal pathway [23,24]. Thus, we performed coimmunoprecipitation on VSMCs transfected with siRNA of *Zfp36* and *Gbp2*, which showed that *Zfp36* knockdown markedly decreased the combination between YAP1 and TEAD1 and promoted YAP1/KLF4 complex formation simultaneously. Besides, knockdown of *Gbp2* increased the TEAD1 expression, restoring the YAP1/TEAD1 complex and inhibiting YAP1/KLF4 complex formation (Fig. S7A and B). Chromatin immunoprecipitation (ChIP) was performed to verify the effect of ZFP36/GBP2 on the transcription activities mediated by the YAP1/TEAD1 complex and the YAP1/KLF4 complex. Results showed that *Zfp36* knockdown decreased the combination of YAP1 to the TEAD1 binding site within the *Tagln* promoter and promoted the combination of YAP1 to the KLF4 binding site within the *Tagln* promoter, while GBP2 knockdown diminished these effects (Fig. S7C), further demonstrating that ZFP36 deficiency led to GBP2 up-regulation and YAP1/KLF4 complex formation, and eventually disrupting phenotypic related gene expression. Besides, we observed significantly increased GBP2 expression and decreased TEAD1 expression in human AAA tissue, while YAP1showed no difference (Fig. S7D).

Taken together, our results demonstrated that ZFP36 manipulated VSMC phenotypic switch via regulating GBP2/YAP1/TEAD1 signaling; ZFP36 knockdown promoted GBP2 expression and TEAD1 degradation, which led to YAP1 dissociating from TEAD1 and bonding to KLF4, forming the YAP1/KLF4 complex and causing VSMC phenotypic switch and AAA progression.

### Dex transcriptionally activates ZFP36 via promoting glucocorticoid receptor nuclear translocation

**Fig. 7.**
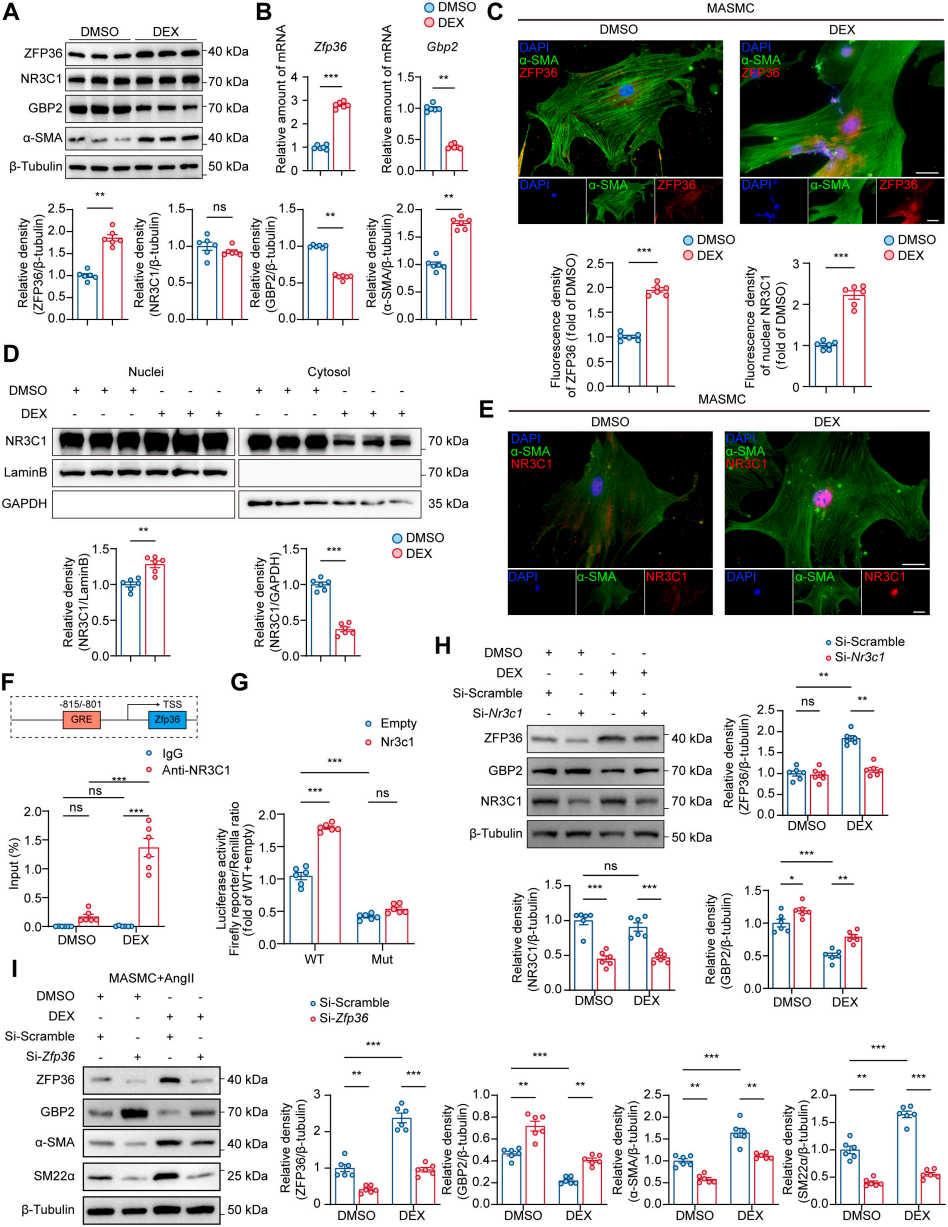
Dexamethasone transcriptionally activates ZFP36 via promoting glucocorticoid receptor nuclear translocation. (A and B) Protein levels and relative mRNA levels of VSMCs treated with DMSO or dexamethasone (0.5 μM) for 48 h (*n* = 6 per group). (C) Representative images of NR3C1 expression by IF staining of VSMCs treated with DMSO or dexamethasone (0.5 μM) for 48 h and quantification (*n* = 6 per group). Scale bar indicates 20 μm. (D) Nuclear and cytoplasmic protein levels of VSMCs treated with DMSO or dexamethasone (0.5 μM) for 48 h (*n* = 6 per group). (E) Representative images of ZFP36 expression by IF staining of VSMCs treated with DMSO or dexamethasone (0.5 μM) for 48 h (*n* = 6 per group) and quantification. Scale bar indicates 20 μm (*n* = 6 per group). Statistical analyses of (A), (B), (C), (D), and (E) were analyzed by unpaired *t* test. (F) ChIP was performed with antibodies to NR3C1, and the target promoter region of *Zfp36* was amplified by qPCR (*n* = 6 per group). (G) Luciferase reporter assay. Firefly luciferase activity was normalized to Renilla activity and expressed as relative luciferase activity (*n* = 6 per group). (H) Protein levels of VSMCs treated with DMSO or dexamethasone (0.5 μM) for 48 h after transfecting with Si-Scramble or Si-*Nr3c1* (*n* = 6 per group). (I) Protein levels of VSMCs treated with DMSO or dexamethasone (0.5 μM) for 48 h after transfecting with Si-Scramble or Si-*Zfp36* (*n* = 6 per group). Statistical analyses of (F), (G), (H), and (I) were analyzed by 2-way ANOVA following Tukey’s multiple comparisons test. ns indicates not significant; *P < 0.05; **P < 0.01; ***P < 0.001.

Previous studies suggested that Dex could be a potent agonist of ZFP36 [25–27]; we first test the effect of Dex on VSMCs. WB and RT-qPCR results showed that treating VSMCs with Dex of 0.5 μM for 48 h significantly activated ZFP36 (Fig. S8A and B). We also examined the downstream of ZFP36 after Dex treatment; WB and RT-qPCR results indicated that Dex up-regulated ZFP36 and α-SMA but down-regulated GBP2 (Fig. 7A and B). IF staining also verified the activation effect of Dex on ZFP36 in VSMCs (Fig. 7C). Glucocorticoid receptor (also known as NR3C1, nuclear receptor subfamily 3 group C member 1) is the classic target of Dex; WB results of total protein showed no difference in NR3C1 after Dex treatment (Fig. 7A). Thus, we separated nuclear protein and cytoplasmic protein followed by WB, and the results showed that Dex treatment significantly increased nuclear NR3C1 but decreased cytoplasmic NR3C1 (Fig. 7D). IF staining indicated the strong nuclear location of NR3C1 after Dex treatment (Fig. 7E). Of interest, we found that AngII could decrease nuclear NR3C1 level (Fig. S9A), and IF staining showed that NR3C1 translocated from the nucleus into the cytoplasm (Fig. S9B). These results suggested that AngII led to the down-regulation of ZFP36 in an NR3C1-dependent manner.

NR3C1 is a well-known transcriptional factor; thus, we predicted binding sites of NR3C1 within the *Zfp36* promoter based on the Transcription Factor Database (http://jaspar.genereg.net). ChIP was performed on the 4 predicted sites, qPCR results showed that NR3C1 mainly binds to the glucocorticoid receptor response element (GRE) between −815 and −801 bp within the *Zfp36* promoter, and Dex treatment could strengthen the binding (Fig. 7F); the 3 other sites showed weak binding and no changes after Dex treatment (Fig. S10). Dual luciferase reporter assay showed that NR3C1 significantly increased the luciferase activity of the promoter reporter containing the GRE but had no effect on the luciferase activity of the promoter with a mutated binding site (Fig. 7G). The above results proved that Dex treatment induced *Zfp36* transcriptional activation via promoting NR3C1 nuclear translocation.

We further verified that the effect of Dex relies on NR3C1 by transfecting siRNA of *Nr3c1* into VSMCs treated with Dex, and WB results showed that NR3C1 knockdown fully abolished Dex’s activation on ZFP36 (Fig. 7H). WB results also demonstrated that Dex has an effect on promoting contractile protein expression via ZFP36 up-regulation (Fig. 7I), and knockdown of *Zfp36* fully abolished its role. Taken together, our results suggested that Dex promotes contractile phenotype of VSMCs by promoting NR3C1 nuclear translocation and subsequent ZFP36 transcriptional activation.

### Administration of low-dose Dex prevents AAA formation in a ZFP36-dependent manner

Dex is a classical drug with powerful effects and has been widely used in clinics [28], but its potential in AAA treatment has not been determined. To test the therapeutic effect of Dex on AAA via ZFP36, we first performed in vivo experiments to determine the dosage of Dex. Male C57BL/6J mice were randomly subdivided into 3 groups and subjected to intraperitoneal injection daily for 28 days. WB of aortic tissues indicated that Dex treatment of low dose (10 μg/kg/day) led to a remarkable up-regulation of ZFP36, while medium- and high-dose treatment has moderate effect (Fig. S11A). RT-qPCR results also confirmed that low-dose treatment up-regulated *Zfp36* while high-dose treatment has almost no effect (Fig. S11B). IF staining also indicated the significant up-regulation of ZFP36 of VSMCs in the low-dose group, while high-dose treatment showed weak effects (Fig. S11C). Thus, we performed drug administration experiments based on the tested dosage of Dex.

**Fig. 8.**
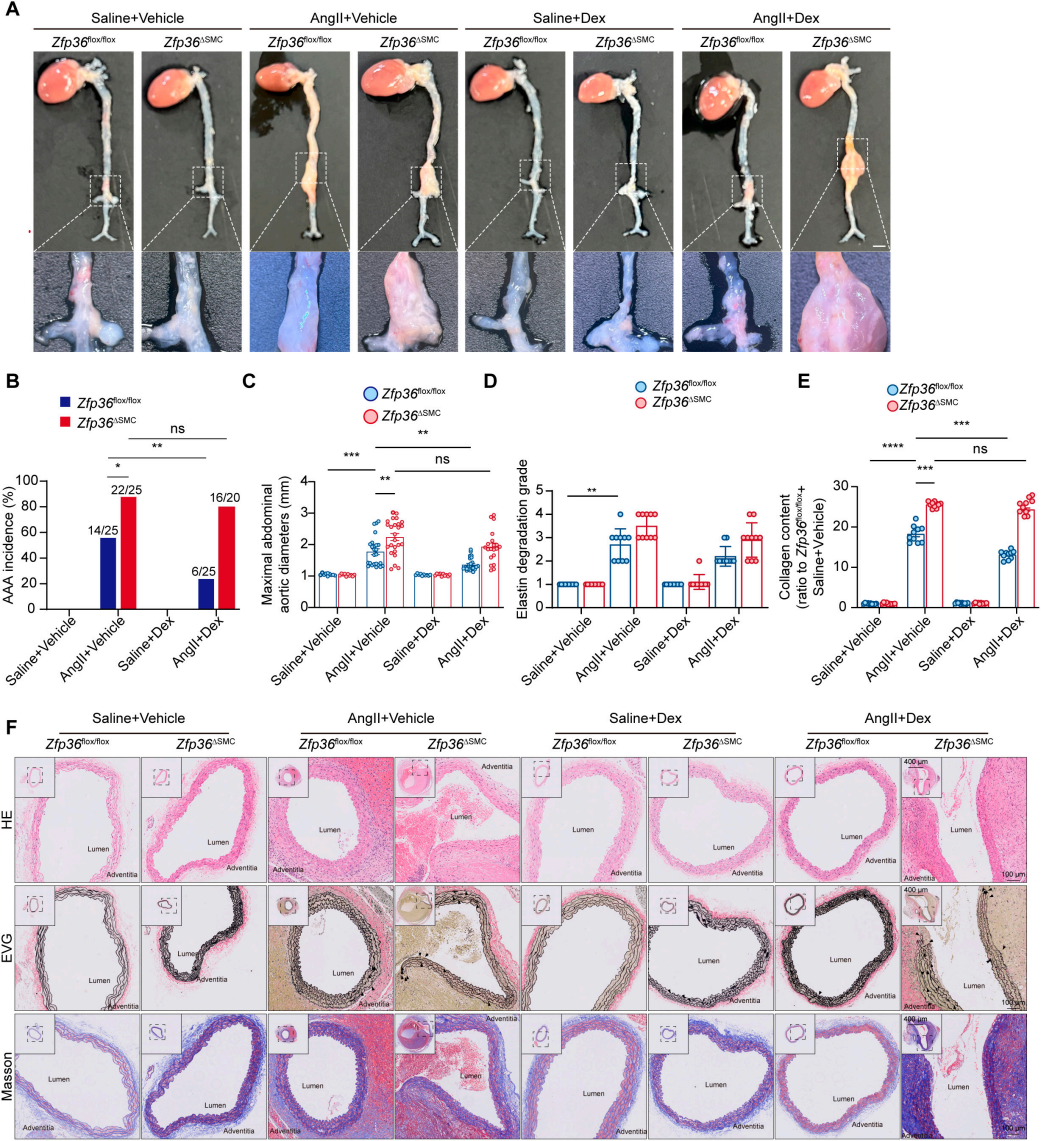
Administration of low-dose dexamethasone prevents AAA formation in a ZFP36-dependent manner. (A) Representative images of macroscopic features of cross-sections of abdominal aortas. Scale bar indicates 2 mm. (B) Incidence of AAA induced by AngII in indicated groups (*n* = 10 for saline administration; *n* = 20 to 25 for AngII administration). Data were analyzed by a Fisher exact test. (C) Quantification of the maximal diameter of suprarenal abdominal aortas (*n* = 11 for saline administration; *n* = 14 for AngII administration). Data were expressed as the mean ± SEM and analyzed by 2-way ANOVA following Tukey’s multiple comparisons test. (D) Grade of elastin degradation (*n* = 10 per group). Data were expressed as median with interquartile range and analyzed by nonparametric Kruskal–Wallis test with Dunn’s post-hoc test. (E) Quantitative analysis of collagen deposition (*n* = 10 per group). Data were expressed as the mean ± SEM and analyzed by 2-way ANOVA following Tukey’s multiple comparisons test. (F) Representative images of HE, Masson, and EVG staining of cross-sections of abdominal aortas. ns indicates not significant; *P < 0.05; **P < 0.01; ***P < 0.001.

As shown in Fig. 8A, we established an AAA model as previously described and started Dex administration after osmotic pump implantation. As we expected, Dex administration markedly inhibited AAA formation; mice infused with AngII showed lower mortality, less AAA incidence, and mean maximal abdominal aortic diameters after Dex treatment (Fig. 8A to C). Of interest, mice lacking ZFP36 in VSMCs could not benefit from Dex; the mortality, AAA incidence, and mean maximal abdominal aortic diameters showed no differences between the *Zfp36*^△SMC^ + AngII + Vehicle group and the *Zfp36*^△SMC^ + AngII + Dex group. These results showed that Dex protected against AAA formation in a ZFP36-dependent manner. We further evaluated the aortic remodeling degree via histology staining (Fig. 8D). Dex treatment markedly reversed aortic remodeling in *Zfp36*^flox/flox^ mice with less elastin breaks and collagen deposition (Fig. 8E and F), and it still had no effects on *Zfp36*^△SMC^ mice, which is consistent with former results. Taken together, our results proved that a low dose of Dex could be an effective strategy in treating AAA via up-regulating ZFP36.

## Discussion

**Fig. 9.**
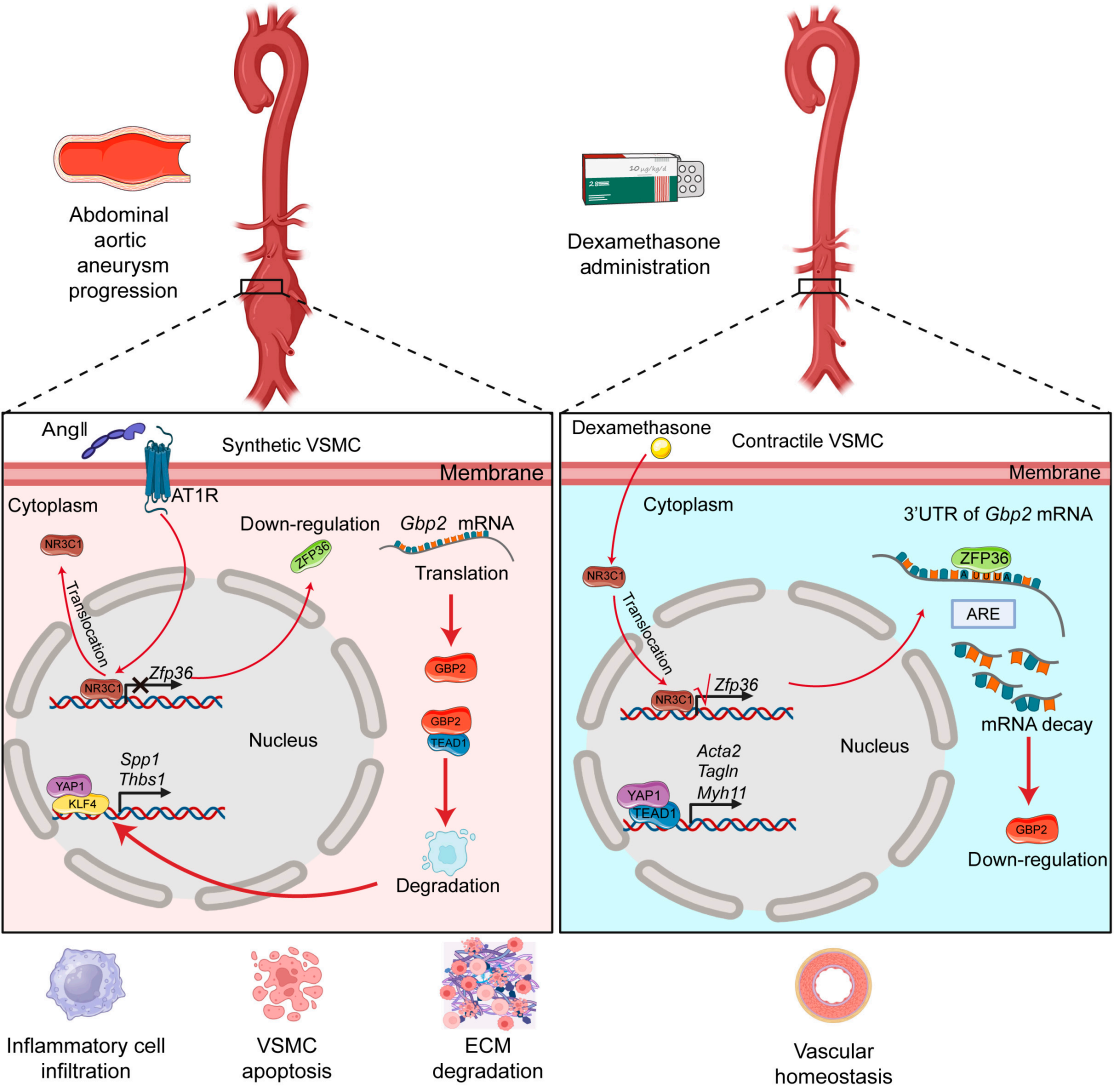
Schematic diagram of the molecular mechanisms. The diagram is created in BioRender (this work is licensed under CC BY 4.0).

In the present study, we discovered that ZFP36 was down-regulated during AAA progression in both human and mice. VSMC-specific ZFP36 deficiency accelerated AAA formation while VSMC-specific overexpressing ZFP36 inhibited AAA progression induced by AngII. Mechanistically, AngII increased NR3C1 cytoplasmic location and thus repressed ZFP36 transcription, which caused GBP2 up-regulation, TEAD1 degradation, and YAP1/KLF4 complex formation, eventually leading to VSMC phenotypic switch and AAA progression. Utilization of Dex activated ZFP36 expression via increasing NR3C1 nuclear location. Up-regulated ZFP36 bonded to 3′UTR of *Gbp2* mRNA and promoted its decay, inhibiting YAP1/KLF4 complex formation and VSMC phenotypic switch (Fig. 9). Collectively, our findings reveal the pivotal role of ZFP36 in mediating VSMC function and maintaining vascular homeostasis, and thereby expand the therapeutic usage of Dex, offering a novel strategy for AAA treatment.

ZFP36, also known as tristetroprolin, is widely involved in inflammatory diseases via promoting specific mRNA decay [29]. Despite its well-known anti-inflammatory effects, ZFP36 also participates in the regulation of multiple life activities including metabolism, lipophagy, and necroptosis [30,31]. Recently, the beneficial roles of ZFP36 in the cardiovascular system have been reported [12]. ZFP36 was first identified to be highly expressed in circulating monocytes and plaque macrophages as the transcriptional regulator [32]. It was also verified that ZFP36 could bind to the 3′UTR of *Tnfa* and *Il1b* mRNAs and thereby suppressed inflammation in THP-1 cells [33]. Subsequently, ZFP36 was proven to inhibit macrophage-derived foam cell formation via promoting CD36 mRNA decay [13]. In vitro and in vivo experiments confirmed that ZFP36 expressed in aortic endothelial cells. Besides inhibiting inflammation, ZFP36 was also proven to regulate reactive oxygen and nitrogen species and mediate vasorelaxation [34]. To date, the involvement between ZFP36 and VSMC has not been poorly understood. The present study first generated VSMC-specific *Zfp36* deletion mice and reported the beneficial role of ZFP36 in maintaining the VSMC phenotype and vascular homeostasis via promoting *Gbp2* mRNA decay, providing novel understanding of ZFP36 function.

VSMC, the predominant cell type in the aortic wall, plays a vital role in regulating vasomotor tone of blood vessels [35]. During aortic remodeling, VSMCs respond to arterial injury by switching from a contractile phenotype (physiological state) to a synthetic phenotype (pathological state), initiating ECM degradation, inflammation, and AAA formation [36]. Recently, the VSMCs of pathological state were further classified into various subtypes [37]. Besides the classical synthetic phenotype, various phenotypes are characterized by different markers, including osteochondrogenic VSMCs (Runx2 and Sox9), myofibroblast-like VSMCs (fibronectin, collagen 1, and S100a4), macrophage-like VSMCs (CD11b, CD68, and Lgals3), adipocyte-like VSMCs (Ucp1), and mesenchymal-like VSMCs (Sca1) [38]. These dedifferentiated VSMCs lost expression of contractile proteins, shared features with tumor cells, and thus driven aortic diseases such as atherosclerosis [39].

Numerous studies proved that transcription factors (TFs) such as TEAD1 are core components of the regulatory network of VSMC phenotypic switch [40]. TEAD1 and its coactivator YAP1 are the classic members of the Hippo pathway, which have been demonstrated to be involved in VSMC phenotypic switch regulation [41]. VSMC-specific *Yap1* deletion induced VSMC dysfunction and spontaneous aneurysm [42], while VSMC-specific deletion of *Tead1* led to embryonic lethality with impaired vascular wall generation [43]. Of interest, it has also been reported that TEAD1 or YAP1 accelerated injury-induced VSMC proliferation and vascular stenosis [44]. Other studies proved the vital role of TEAD1 in promoting VSMC differentiation via interacting with VGLL4 in pluripotent stem cells [45,46]. Taken together, it is convincing that YAP1/TEAD1 plays an important but diversified role in VSMC phenotypic switch regulation, and the actual effects on VSMCs depend on the interaction factors. In this study, we found that ZFP36 deficiency led to GBP2 up-regulation and subsequent TEAD1 degradation. Considering that KLF4 is a well-known TF related to VSMC phenotypic switch, we examined its interaction with YAP1 and found increased binding between YAP1 and KLF4 after *Zfp36* knockdown, which suggested that KLF4 replaced TEAD1 and formed a complex with YAP1 to promote VSMC phenotypic switch. Taken together, our results provided novel insights into the plural roles of YAP1/TEAD1 in regulating VSMC phenotypic switch. Besides, our research establishes GBP2, an inflammation-related gene, as a key regulator of VSMC phenotypic switch via the YAP1/TEAD1 pathway. This finding provides mechanistic insight into AAA pathogenesis, underscoring how GBP2-induced VSMC dysfunction promotes AAA progression. Furthermore, our results demonstrate that pharmacological inhibition of this phenotypic switch represents a promising therapeutic strategy for AAA.

Dex is one of the most commonly used drugs in clinics for treating allergic and auto-immune diseases [47]. Dex is favorable for doctors because of its powerful effects; numerous researchers have been exploring its novel utilization for decades. According to ClinicalTrials.gov, the number of registered clinical trials about Dex has surpassed 4,000, and the applicable field of Dex has been expanded in recent years [48,49], which suggested that Dex is a good alternative for developing medication. The therapeutic potential of Dex and other corticosteroids in the management of aneurysmal diseases has long been a subject of considerable interest. Clinical evidence has confirmed benefits from Dex in certain subarachnoid hemorrhage patients following open surgery [50]. This is supported by preclinical findings demonstrating that Dex exerts protective effects in aneurysmal models by mitigating inflammation and improving collagen synthesis [51,52]. Furthermore, multiple studies have highlighted the promising therapeutic prospects of Dex in thoracic aortic aneurysms [53,54]. However, the underlying mechanisms and its specific role on AAA remained unclear. We performed in vivo experiments based on the dosage previously verified and the dosage used in clinics, and found that low-dose (10 μg/kg/day) Dex administration could exert therapeutic effects on AAA via up-regulating ZFP36. Side effects are the main problem that limits Dex utilization. The dose we adopted (10 μg/kg/day) on mice is identical to approximately 0.076 mg/day on humans, which is about 10% of the clinical therapeutic dose (0.75 mg/day), which could minimize side effects. We believe that the successful application of Dex for AAA treatment hinges critically on determining its optimal dosage and route of administration. Glucocorticoids such as Dex have complicated usage depending on the disease context. In critical conditions like septic shock, short-term high-dose therapy is typically employed. For diseases such as infections and transplant rejection, short- to medium-term treatment is applied, with gradual tapering before ending medication. For treating chronic immune diseases with medication exceeding 3 months, maintenance therapy is initiated first. Prior to cessation, the medication is transitioned to an every-other-day regimen and then gradually phased out [55]. The administration of Dex is highly versatile, encompassing oral, intramuscular injection, intravenous injection, or drip for systemic delivery, as well as localized methods such as inhalation, local injection, or topical application [56]. In this study, intraperitoneal injection was employed, suggesting that low-dose Dex treatment over 28 days can mitigate AAA progression in mice. However, before Dex could be applied in clinical practice for AAA, further research is essential. Key questions include how to effectively taper the dosage following maintenance therapy in patients with progressive AAA, whether high-dose Dex therapy could benefit those with ruptured AAA, and how to determine the optimal administration method for different patient populations.

Currently, the primary strategy for AAA consists of surgical interventions, including open repair surgery and endovascular aneurysm repair [2]. However, for small aneurysms that do not meet surgical criteria, effective treatment options remain limited. Previous studies have demonstrated that pharmacological therapies (such as beta-blockers, lipid-lowering agents, and anti-platelet drugs) can modestly slow AAA progression but are unable to reverse the disease process [57–59]. Furthermore, the use of anti-platelet and anti-hypertensive medications becomes constrained in cases where AAA rupture necessitates emergency surgery. These limitations underscore the critical need for developing effective pharmacological treatments for AAA. Dex is a potent glucocorticoid with well-established anti-inflammatory properties, reducing vascular damage through inflammation suppression [60,61]. This suggests its potential utility as an adjunctive therapy for surgical patients. Our research provides further evidence that low-dose Dex administration prevents AAA via improving VSMC function, indicating its potential applicability even for treating small AAA.

Our study has certain limitations. First, we chose GBP2 as the primary downstream target of ZFP36 for in-depth investigation based on its high expression and substantial variation in AngII-treated VSMCs and aneurysm. It is nevertheless plausible that other GBP family members, such as GBP10, play important roles, a possibility that merits future verification. Furthermore, while our study demonstrates the therapeutic potential of low-dose Dex in mitigating AngII-induced VSMC phenotypic switching and AAA progression in preclinical models, these findings are derived solely from cellular and animal experiments. The lack of validation in large-scale clinical cohorts represents a major limitation. Therefore, future work is paramount to determine the optimal dosage and ideal administration route, and to address potential off-target effects prior to clinical translation.

In conclusion, we demonstrated that ZFP36 exerted a protective role against VSMC phenotypic switch and AAA formation. The finding of ZFP36/GBP2/YAP1/TEAD1 signaling provides novel insights into the underlying mechanisms of AAA progression. Additionally, we verified the activation effect of Dex on ZFP36 and its therapeutic potential in AAA treatment, which provides a new strategy for clinical utility.

## Materials and Methods

### Animals

The protocols of animal experiments in this study were approved by the Ethics Committee of Qilu Hospital of Shandong University (DWLL-2024-398), and all procedures were conducted following the National Institutes of Health Guidelines for the Care and Use of Laboratory Animals. All mice used in this study were housed under specific pathogen-free conditions at a temperature of 23 °C with 60% relative humidity and a 12-h dark/light cycle. For AAA modeling, mice were first subjected to 2.5% isoflurane to induce anesthesia. During the process of mini pump implantation, mice were continuously anesthetized with 1.5% isoflurane. At the termination of experiments, mice were euthanized by intravenous injection of a lethal dosage of pentobarbital sodium (150 mg/kg). Animal experiments were conducted independently by 2 investigators who were blinded to the group assignments. Randomization was performed using the RAND function in Excel to generate group allocations based on body weight, with the assignment information concealed from the experimenters throughout the study. The sample size for the mouse aortic aneurysm experiments was determined a priori using PASS software, based on preliminary data from similar AAA models in our laboratory. A minimum of 8 animals per group was required to achieve 90% power (*β* = 0.1) for detecting significant differences in maximal aortic diameter at a significance level of *α* = 0.05. Each mouse was treated as an independent experimental unit.

The mice used in this study are on a C57BL/6 background. *Zfp36*^flox/flox^ mice were kindly given by Prof. Pavel Kovarik (Max Perutz Labs, University of Vienna, Vienna BioCenter, Vienna, Austria) and *Tagln*-Cre mice were purchased from Cyagen Biosciences. VSMC-specific *Zfp36*-deficient mice (*Zfp36*^flox/flox^/*Tagln*-Cre, *Zfp36*^△SMC^) were generated by crossbreeding *Zfp36*^flox/flox^ mice and *Tagln*-Cre mice [62,63]. Male mice aged 8 weeks were used in this study. A single injection of recombinant type 8 adeno-associated virus of murine proprotein convertase subtilisin/kexin type 9 mutants (rAAV8/D377Y-mPCSK9, 2 × 10^11^ vg) was performed to each mouse 2 weeks prior to osmotic pump implantation combined with the Paigen diet to induce hypercholesterolemia as previously described. The AngII-induced AAA models were established as previously reported [15]. Briefly, the ALZET osmotic pumps (model 2004, Durect, Cupertino, CA, USA) were infused with AngII (Sigma-Aldrich, St. Louis, MO, USA) or saline. Mice were anesthetized with pentobarbital sodium before subjected to osmotic pump implantation. The delivery of AngII (MCE, HY-13948, 1,000 ng/kg/min) or saline lasts 28 days. Aneurysm progression was evaluated by ultrasound imaging and the severity of AAA was determined by measuring the maximal aortic diameters using a caliper at sacrifice. Macroscopic photos of aortas were taken by a Nikon camera and periadventitial tissues were removed before taking pictures. We used the well-accepted criterion to define AAA: the maximal aortic diameter of an aneurysm is at least 1.5 times the aortic diameter of mice infused with normal saline. The elastase-induced AAA models were established as previously reported [64]. Briefly, male mice were placed in a supine position after anesthesia. Following hair removal and disinfection, a longitudinal laparotomy was performed to expose the abdominal cavity. The infrarenal abdominal aorta was then carefully isolated via blunt dissection. The exposed aortic segment was wrapped in cotton pads saturated with 30 μl of porcine pancreatic elastase (PPE group, total activity: 1.8 units) or heat-inactivated elastase (Sham group). The aorta was incubated for 30 min. Subsequently, the cotton pads were removed, and the abdominal cavity was irrigated with sterile saline. Finally, the incision was sutured to close the abdominal cavity.

To determine the effect of ZFP36 overexpressing on AAA formation, male C57BL/6J mice aged 8 weeks were injected with rAAV8/D377Y-mPCSK9 (2 × 10^11^ vg) first and fed with Paigen diet. Three days later, mice were randomly divided into 2 groups and injected with AAV2-Ctrl or AAV2-*Zfp36* under *Tagln* promoter (2 × 10^11^ vg). The AngII-induced AAA model was established 2 weeks after AAV injection. A rescue experiment was performed using *Zfp36*^flox/flox^ mice and *Zfp36*^△SMC^ mice. Male C57BL/6J mice aged 8 weeks were injected with rAAV8/D377Y-mPCSK9 (2 × 10^11^ vg) first and fed with Paigen diet. Three days later, mice were randomly divided into 2 groups and injected with pAAV-SM22ap-MCS-mCherrymiR30shRNA (NC)-WPRE (AAV-ShNC) or pAAV-SM22ap-MCS-mCherrymiR30shRNA (Gbp2)-WPRE (AAV-Sh*Gbp2*) (2 × 10^11^ vg). The AngII-induced AAA model was established 2 weeks after AAV injection.

Male C57BL/6J mice aged 8 weeks were randomly divided into 3 groups and subjected to Dex (MCE, HY-14648, dissolved in saline; low dose: 10 μg/kg/day; high dose: 50 μg/kg/day) or vehicle (saline) intraperitoneal injection daily for 28 days. To determine whether Dex exerts a therapeutic effect on AAA in a ZFP36-dependent manner, male *Zfp36*^△SMC^ mice and littermate *Zfp36*^flox/flox^ mice aged 8 weeks were used. The AAA model was established as previously described. Dex administration was performed after pump implantation, and mice received intraperitoneal injection of Dex (dissolved in saline; 10 μg/kg/day) or saline daily for 28 days.

For histological analysis, aortas were flushed with saline and subsequently submerged into paraformaldehyde for fixation. For molecular experiments, aortas were quickly removed, flushed, and then stored in liquid nitrogen.

### Clinical study participants

All human samples used in this study were obtained following protocols approved by the Ethical Committee of Qilu Hospital of Shandong University (KYLL-2022-942). The study was performed according to the criteria set by the Declaration of Helsinki (2013). Human AAA tissue samples were obtained from patients who underwent open surgical repair. Healthy abdominal aorta samples from brain-dead organ donor patients were used as control. All study participants were recruited after providing informed consent and with approval by the Ethics Committee of Qilu Hospital of Shandong University.

### Statistical analysis

All statistical analyses were performed using GraphPad Prism 10. For statistical comparisons, Shapiro–Wilk test was performed initially to evaluate data for normality. Normally distributed data are presented as mean ± standard error of the mean (SEM) while nonnormally distributed data are presented as median and interquartile range. A *P* value < 0.05 was considered statistically significant.

## Data Availability

The data of this article will be made available upon reasonable request from the corresponding author.

## Supplementary Materials

Supplementary 1 Expanded Methods Table S1 Figs. S1 to S11 Reference [65]
