# Supplementary material for: ZFP36 Protects against Abdominal Aortic Aneurysm Formation by Regulating Vascular Smooth Muscle Phenotypic Switch
Source: Research (Wash D C). 2026 Jan 16;9:1078. doi: 10.34133/research.1078 (PMC12808826; doi:10.34133/research.1078)
Supplement: Supplementary 1 — Expanded Methods Table S1 Figs. S1 to S11 Reference [65] [file research.1078.f1.doc]

**SUPPLEMENTAL MATERIAL**

**Expanded Methods**

**Ultrasound Imaging**

Mice were placed on the heating panel under anesthesia with isoflurane. Before detecting, the abdominal hair was removed using depilatory cream. Images were obtained by the *Vevo* 2100 high resolution imaging system (Visual Sonics, Inc., Toronto, Canada). The images of abdominal aortas were recorded on both long axis and short axis.

**Production and Injection of AAV**

The adeno-associated viruses (AAVs) used in this study including rAAV8/D377Y-mPCSK9, AAV2-Ctrl and AAV2-*Zfp36* were constructed and packaged by Vigene Bioscience (Jinan, China). The AAVs including pAAV-SM22ap-MCS-mCherrymiR30shRNA (NC)-WPRE (AAV-ShNC) and pAAV-SM22ap-MCS-mCherrymiR30shRNA (Gbp2)-WPRE (AAV-Sh*Gbp2*) constructed and packaged by OBiO Technology (Shanghai) Corp.,Ltd. Mice were injected with AAV (2×10^11^ vg) at age of 8 weeks and subjected to correspondent operations 2 weeks after injection.

**Histological Assay and Immunofluorescent Staining**

Fixed mouse aortas were embedded in paraffin and cut into 5μm sections for pathological staining or immunofluorescent (IF) staining. Hematoxylin and eosin (H&E), Masson’s trichrome, and elastic Van Gieson staining were performed to evaluate morphological changes, collagen deposition and elastin break respectively. The extent of elastin degradation in EVG-stained sections was assessed using a semi-quantitative 4-grade scale established by Satoh et al.[65], with the following criteria: Grade 1, No degradation; Grade 2, Mild degradation; Grade 3, Severe degradation; Grade 4, Aortic rupture.

For IF staining, selected sections underwent gradient rehydration, antigen repair and blocking were incubated with primary antibodies overnight at 4°C. Antibodies used in IF staining are listed as follows: α-SMA (1:200, Proteintech, 67735-1-Ig), ZFP36 (1:100, Proteintech, 12737-1-AP), GBP2 (1:100, Proteintech, 11854-1-AP), NR3C1 (1:100, Proteintech, 24050-1-AP). The other day, sections were washed with PBS for 3 times and incubated with Alexa Fluor 488-/555-conjugated secondary antibodies (1:1000, Invitrogen, USA) at room temperature in a humid chamber for an hour. DAPI (P0131, Beyotime Biotechnology, Shanghai, China) was added to visualize the nuclei before sealing. Images were captured by electric microscope (DS-Ri2, Nikon, Japan). Dual-labeled immunofluorescence images were processed and quantified using ImageJ software. Following multi-channel merging, background fluorescence correction, and image registration, dual-positive regions were identified by performing logical operation between two single-positive regions via the Image Calculator function. The Analyze Particles function was then used to quantify the pixel areas of dual-positive regions and the total tissue area. Data from multiple fields of view were exported to GraphPad Prism software, and the percentage of dual-positive area relative to the total area was adopted as the primary metric for intergroup comparisons.

**Assessment of VSMCs Apoptosis**

To evaluate the levels of VSMCs apoptosis in abdominal aortas, terminal deoxynucleotidyl transferase dUTP nick-end labeling (TUNEL) assay was performed, using an *In Situ* Cell Death Detection Kit, TMR red (12156792910; Roche, Germany). VSMCs were labeled by the anti-α-SMA antibody and Alexa Fluor 488-conjugated secondary antibodies (1:1000, Invitrogen, USA), and the nuclei were visualized by DAPI. Images were captured by electric microscope (DS-Ri2, Nikon, Japan). For TUNEL assay analysis, after image acquisition, the numbers of dual-positive VSMCs (SMA⁺/TUNEL⁺) and total VSMCs (SMA⁺) were counted based on DAPI-defined nuclei. The apoptotic rate was calculated as the ratio of dual-positive cells to total VSMCs and subjected to statistical analysis in GraphPad Prism.

**Gelatin Zymography Assay**

To evaluate the MMPs activity, proteins of aortic tissues were electrophoresed in SDS-PAGE gels that contained 1 mg/ml gelatin. Gels were washed in 2.5% Triton X-100 and incubated for 48h (37°C) in zymography buffer [50 mmol/L Tris (pH 8.0), 10 mmol/L CaCl_2_, and 0.05% Brij35]. Subsequently, the gels were stained with Coomassie brilliant blue and decolorized using wash buffer containing methanol and acetic acid.

**Biochemical Analysis**

All mice were fasted overnight and weighed before collecting blood. The plasm was separated and measured for total cholesterol and total triglycerides using commercial kits (Nanjing Jiancheng Technology, A110-1-1, A111-1-1) following manufacturer’s instructions.

**Primary VSMCs Isolation and Culture**

Mouse primary vascular smooth muscle cells (VSMCs) were isolated by enzymatically separation. C57BL/6J mice at age of 6-8 weeks were euthanized and the aorta was quickly removed. The adventitial layer and surrounding tissue were removed smoothly using dissecting microscopes. The remaining abdominal aortas were fully cut into small pieces, resuspended with Type 2 collagenase (1 mg/ml, dissolved in DMEM/F12 culture medium, LS004176, Worthington Biochemistry, Lakewood, NJ, USA), moved into 6 cm dishes and kept in culture incubator for 8h at 37°C. Equal volume of complete medium (DMEM/F12 culture medium containing 10% FBS, Gibco, USA) was added to the dishes to terminate the digestion. Digested tissues were collected by centrifugation at 1,000 g for 5 min, and were suspended with fresh culture medium and seed into 6 cm dishes. The passaging of VSMCs were performed at the confluence of 80%. VSMCs of passage 2-4 were used in this study.

**Western blotting analysis**

Total protein of cells and aortic tissues were extracted using RIPA buffer added protease inhibitor cocktail (AR0102, Boster Biological Technology, Wuhan, China). The nuclear and cytoplasmic proteins were separated using commercial kits following manufacturer’s instructions (P0027, Beyotime Biotechnology, Shanghai, China). Denatured proteins were separated by electrophoresis using SDS-polyacrylamide gels and transferred to PVDF membranes (0.45μm, Millipore). Members were blocked with 5% skimmed milk at room temperature for 1 hour and incubated with primary antibodies at 4°C overnight. Antibodies used in the experiments are listed as follows: α-SMA (1:1000, Proteintech, 67735-1-Ig), ZFP36 (1:1000, Proteintech, 12737-1-AP), GBP2 (1:1000, Proteintech, 11854-1-AP), NR3C1 (1:1000, Proteintech, 24050-1-AP), β-Tubulin (1:1000, Proteintech, 10068-1-AP), MMP2 (1:1000, Cell Signaling Technology, 87809), Bax (1:1000, Cell Signaling Technology, 2772), Bcl2 (1:1000, Cell Signaling Technology, 3498), Caspase-3 (1:1000, Cell Signaling Technology, 14220), Cleaved Caspase-3 (1:1000, Cell Signaling Technology, 9664), MYH11 (1:1000, Proteintech, 21404-1-AP), SM22α (1:1000, Proteintech, 10493-1-AP),TSP-1 (1:1000, Proteintech,18304-1-AP), KLF4 (1:1000, Proteintech, 11880-1-AP), OPN (1:1000, Proteintech, 22952-1-AP), TEAD1 (1:1000, Cell Signaling Technology, 12292), YAP1 (1:1000, Proteintech, 13584-1-AP), LaminB (1:1000, Proteintech, 12987-1-AP), GAPDH (1:5000, Proteintech, 60004-1-Ig). Peroxidase AffiniPure™ Goat Anti-Mouse IgG (H+L) (115-035-003), Peroxidase AffiniPure™ Goat Anti-Rabbit IgG (H+L) (111-035-003) (1:10000, Jackson ImmunoResearch Europe Ltd.) and enhanced chemiluminescence (Millipore, Temecula, CA) were used for detecting bound antibodies. The Amersham Imager 680 (GE Healthcare Life Sciences) and Tanon Chemi 5200 Multi automatic luminescence imaging system (Tanon, Shanghai, China) were used for chemiluminescence.

**Reverse transcription-quantitative polymerase chain reaction (RT-qPCR)**

Total RNA of cells and aortic tissues were extracted using RNAiso Plus (9109, Takara) following manufacturer’s instructions. 1μg RNA of each sample was reversely transcribed into cDNA using HiScript IV RT SuperMix for qPCR (R423-01, Vazyme, Nanjing, China). RT-qPCR was performed using Taq Pro Universal SYBR qPCR Master Mix (Q712-03, Vazyme, Nanjing, China) following manufacturer’s instructions. Primers used in the experiments are listed in Table S1. LightCycler® 480 Instrument II (Roche LifeScience) was used for RT-qPCR experiments and data analysis.

**Chromatin Immunoprecipitation (ChIP) Assay**

ChIP assay was performed using the SimpleChIP enzymatic ChIP kit (#9003, Cell Signaling Technology) according to the manufacturer's instructions. Briefly. VSMCs were seed into the 15cm dishes and treated with DMSO or Dexamethasone (0.5μM) for 48h, then cross-linked by formaldehyde for 10 min. Glycine solution was added to stop cross-link. Cells were lysed and sonicated to produce DNA fragment. The 2% of supernatants were used as the chromatin input before adding antibodies. Antibodies (NR3C1, Proteintech, 24050-1-AP, 1:50) and negative control (Normal Rabbit IgG, CST, 2729) were added into solution and incubated at 4°C overnight. 20μl Pierce Proteins G Magnetic Beads (88848, Thermo Fisher Scientific, Waltham, MA, USA) was added in each sample for immunoprecipitation. After washing, de-crosslinking, eluting and purifying, the purified DNA was used for qPCR. The primers used in ChIP-qPCR were listed in Table S1.

In another set of experiments, VSMCs were prepared as previously described. After transfecting, VSMCs were cross-linked by formaldehyde for 10 min. Glycine solution was added to stop cross-link. Cells were lysed and sonicated to produce DNA fragment. The 2% of supernatants were used as the chromatin input before adding antibodies. Antibodies (YAP1, Proteintech, 13584-1-AP, 1:50) and negative control (Normal Rabbit IgG, CST, 2729) were added into solution and incubated at 4°C overnight. 20μl Pierce Proteins G Magnetic Beads (88848, Thermo Fisher Scientific, Waltham, MA, USA) was added in each sample for immunoprecipitation. After washing, de-crosslinking, eluting and purifying, the purified DNA was used for qPCR. The primers used in ChIP-qPCR were listed in Table S1.

**Co-Immunoprecipitation Assay**

Co-Immunoprecipitation Assay was performed to verify the interaction between TEAD1 and GBP2. First, the Flag-GBP2 plasmid and HA-TEAD1 plasmid were constructed by Vigene Bioscience (Jinan, China). HEK-293T cells were seeded into 6 cm dishes and transfected with Flag-GBP2 plasmid and HA-TEAD1 plasmid using Lipo8000 transfection reagent (C0533, Beyotime Biotechnology, Shanghai, China) following manufacturer's instructions. The proteins were extracted using NP-40 lysis buffer added protease inhibitor cocktail (AR0107, Boster Biological Technology, Wuhan, China). Antibodies (Monoclonal ANTI-FLAG® M2 antibody, Sigma-Aldrich, F1804; HA-Tag, Cell Signaling Technology, 3724) and Pierce Proteins G Magnetic Beads (88848, Thermo Fisher Scientific, Waltham, MA, USA) were used for immunoprecipitation. Species- and isotype-matched IgG was used as the negative control. The protein complexes were denatured and separated by SDS-PAGE electrophoresis. The procedure of western blot has been described previously. VeriBlot for IP Detection Reagents (Abcam, ab131366) were used to detect bound primary antibodies to avoid the interferences of IgG heavy chain during chemiluminescence.

**RNA Immunoprecipitation (RIP) Assay**

RNA immunoprecipitation (RIP) assay was performed using Magnetic RIP kit (17-700, Sigma-Aldrich) following manufacturer's instructions. VSMCs were washed with PBS for 3 times, scraped into the 15ml centrifuge tubes and centrifuged at 1500 rpm, 4°C for 5 min to collect cells. Then the cells were re-suspended in RIP lysis buffer. Antibody (ZFP36, Sigma-Aldrich, ABE285) and Protein A/G beads (88848, Thermo Fisher Scientific, Waltham, MA, USA) were used for immunoprecipitation. Species- and isotype- matched IgG was used as the negative control. Immunoprecipitated RNA was reversely transcribed into cDNA after purifying. 1μL of cDNA was applied for PCR. The primers used are listed in Table S1.

**Luciferase Reporter Assays**

The proximal promoter region of *Zfp36* was amplified by PCR using genomic DNA before being cloned into a pGL3-basic vector (Promega, USA). The GRE site-mutant promoter was also fused to a firefly luciferase reporter. HEK-293T cells were co-transfected with plasmids of firefly luciferase reporters of WT or MUT promoters, Renilla luciferase combined with *Nr1h3* expression or empty vector using Lipo8000 transfection reagent (C0533, Beyotime Biotechnology, Shanghai, China). The activity of both firefly and Renilla luciferases were measured using the Dual-Luciferase Reporter Assay system (E1910, Promega) following manufacturer's instructions and firefly luciferase activity was normalized to Renilla activity and expressed as relative luciferase activity. Mus Gbp2 3’UTR-WT and Mus Gbp2 3’UTR-MUT plasmids were also constructed by Boshang Biotech (China). HEK-293T cells were co-transfected with plasmids of firefly luciferase reporters of WT or MUT promoters, Renilla luciferase combined with *Zfp36* expression or empty vector using Lipo8000 transfection reagent. The activity of both firefly and Renilla luciferases were measured using the Dual-Luciferase Reporter Assay system (E1910, Promega) following manufacturer's instructions and firefly luciferase activity was normalized to Renilla activity and expressed as relative luciferase activity.

**mRNA Stability Assay**

VSMCs were seeded into six-well plates and infected with Ad-Ctrl or Ad-*Zfp36* for 48h. Actinomycin D (ActD, Sigma) at 10 μg/ml was added to each well. RNA was extracted at different timepoints, and cellular mRNA level was evaluated using RT-qPCR. RNA stability was described as the percentage of remaining RNA at the recommended time after addition of ActD relative to the RNA content at the 0 h time point.

**Protein Degradation Assay**

VSMCs were seeded into six-well plates and infected with Ad-Ctrl or Ad-*Gbp2* for 48h. Chloroquine (10 μM, MCE, HY-17589A) or MG-132 (10 μM, MCE, HY-13259) were added into wells 4h before sample collecting. The subsequent procedure of western blot was described before.

**RNA Sequencing**

RNA-sequencing was conducted by Sinotech Genomics (Shanghai, China). Briefly, total RNA of aortas from *Zfp36*^△SMC^ mice and *Zfp36*^flox/flox^ mice (n=4 per group) was isolated using TRIzol (15596026, Invitrogen). The data of RNA-sequencing was analyzed by R (V3.2.0). DESeq2 package was used to identify genes differentially expressed between *Zfp36*^△SMC^ mice and *Zfp36*^flox/flox^ mice. The *P*-values were adjusted using the Benjamini and Hochberg approach. The thresholds were set to the false discovery rate (FDR) < 0.1, fold change > 2. Volcano plots were generated using ggplot2 package. The Gene Ontology (GO) enrichment analysis was carried out with DAVID Tools (https://david.ncifcrf.gov/tools.jsp).

**Analysis of Microarray Datasets**

The GEO2R tool (https://www.ncbi.nlm.nih.gov/geo/geo2r/) was used to analyze the three expression datasets (GSE47472). A P-value < 0.05 and an absolute log fold-change (FC) exceeding 1 for the DEGs were used as the cut-off criteria. The list of RNA binding proteins was from RBPmap (V1.20, <http://rbpmap.technion.ac.il/>). The analysis of interacted genes between RBPs and differentially expressed genes in GSE47472 was performed using R (V3.2.0).

**Table S1 Primers sequences used in this study**

| Gene name | Forward Primers | Reverse Primers |
| --- | --- | --- |
| *Larp7* | AAGCAGGTGGACTTCTGGTTT | TCCCATCAGTTGTCAGCTTCT |
| *Zc3h8* | AAATGGAAAGTAAAACGGGACCG | GCTGCATGTCTCACCATCACTA |
| *Rbm15b* | AGGGCGAAGGTGGCTATGT | GCGAGGTGTTAGGTCCGAG |
| *Ppp1r10* | CCGCCACCACCTCCATTCC | CACCTCCAACCATGCCTCCTC |
| *Zgpat* | CTGGAGGAGGAGGAGGAGGAC | TCGGACGCACGCAGAACC |
| *Zfp36* | CTCAGAAAGCGGGCGTTGTC | TGGAGGTAGAACTTGTGGCAGAG |
| *Gapdh* | TGTAGACCATGTAGTTGAGGTCA | TGTAGACCATGTAGTTGAGGTCA |
| *Tnfa* | ATGTCTCAGCCTCTTCTCATTC | GCTTGTCACTCGAATTTTGAGA |
| *Mcp-1* | AGCTGTAGTTTTTGTCACCAAGC | GTGCTGAAGACCTTAGGGCA |
| *Il-1beta* | TGCCACCTTTTGACAGTGATG | TGATGTGCTGCTGCGAGATT |
| *Il-6* | CTCCCAACAGACCTGTCTATAC | CCATTGCACAACTCTTTTCTCA |
| *Tead1* | GAGAGCCCTGCCGAAAACAT | CCTCCGCGTCGTTGTCAAT |
| *Yap1* | TACTGATGCAGGTACTGCGG | TCAGGGATCTCAAAGGAGGAC |
| *Gbp2* | ACCCTGGTTCTGCTTGACACTG | TTGTAGATGAAGGTGCTGCTGAGG |
| *Gbp3* | GAGGCACCCATTTGTCTGGT | CCGTCCTGCAAGACGATTCA |
| *Gbp4* | GGAGAAGCTAACGAAGGAACAA | TTCCACAAGGGAATCACCATTTT |
| *Gbp5* | TCTGTGGATCTCGCCTTTCC | AGGTTGGACGGGTGACAGT |
| *Gbp6* | GTTCCAGGAAGTAACAAAGGCT | ATCCCTAGTCTATTCCCAGTGAC |
| *Gbp7* | TCCTGTGTGCCTAGTGGAAAA | CAAGCGGTTCATCAAGTAGGAT |
| *Gbp8* | ACATCTGTCCATGAACCATGAAG | AAACCGTGATTCTGTCCTGCC |
| *Gbp9* | GGTCACCGGGAATAGACTGG | GGGCCACACTTGTCATAGCA |
| *Gbp10* | CCCAGTGTGCCTCATCAAGAA | GCTACGACCACCACAGGATTTT |
| NR3C1 ChIP for *Zfp36* 1 | ACAGAGAAACCCTGTCTCGAA | AGCCTGGTCTACAGAGTGAGT |
| NR3C1 ChIP for *Zfp36* 2 | AGACTAGGGGGTTGGAAGGG | ATCTAGGCGAGGAAGTGGGT |
| NR3C1 ChIP for *Zfp36* 3 | CTCCACCTCCAAATGCCAAGA | ATCAAAGGCGTGTGTCACCAT |
| NR3C1 ChIP for *Zfp36* 4 | TGGTGACACACGCCTTTGAT | TGTTTTTGTTTTTCGAGACAGGGT |
| YAP1  ChIP for *Tagln* (TEAD1) | TTCTCCCAGCCCAAGAGCTA | CACTCCACCAGGTCCATTCC |
| YAP1  ChIP for *Tagln* (KLF4) | ACGCTCAATCCACTACAGCC | CGGGATTTGGGGAATCCTGT |

**Supplemental Figures and Figure Legends**

**
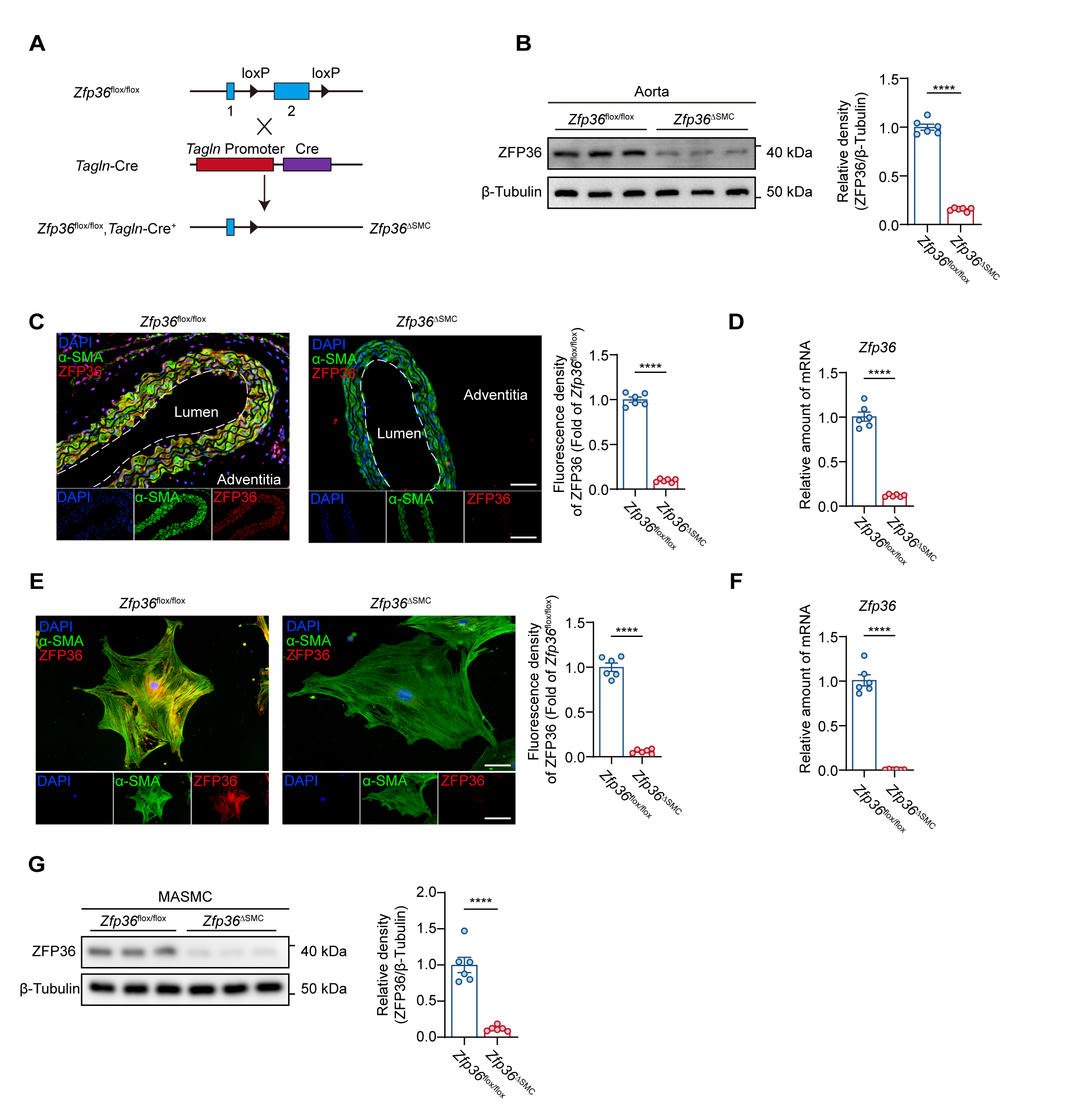
**

**Figure S1. Validation of VSMC-specific *Zfp36* deletion in *Zfp36*^△SMC^ mice.**

**A**, Schematic diagram of VSMC-specific *Zfp36* deficient mice constructing strategy. **B**, Western blot of aortic tissues of *Zfp36*^△SMC^ mice and *Zfp36*^flox/flox^ mice (n=6 per group). **C**, Representative images of immunofluorescence staining of aortic sections from *Zfp36*^△SMC^ mice and *Zfp36*^flox/flox^ mice and the quantification (n=6 per group). Scale bar indicates 50μm. **D**, RT-qPCR of aortic tissues of *Zfp36*^△SMC^ mice and *Zfp36*^flox/flox^ mice (n=6 per group). **E**, Western blot of VSMCs of *Zfp36*^△SMC^ mice and *Zfp36*^flox/flox^ mice (n=6 per group). **F**, Representative images of immunofluorescence staining of VSMCs from *Zfp36*^△SMC^ mice and *Zfp36*^flox/flox^ mice and the quantification (n=6 per group). Scale bar indicates 20μm. **G**, RT-qPCR of VSMCs of *Zfp36*^△SMC^ mice and *Zfp36*^flox/flox^ mice (n=6 per group). Statistical analyses of **A**, **B**, **C**, **D**, **E**, **F** and **G** were analyzed by unpaired *t* test. *****P* < 0.0001。

.


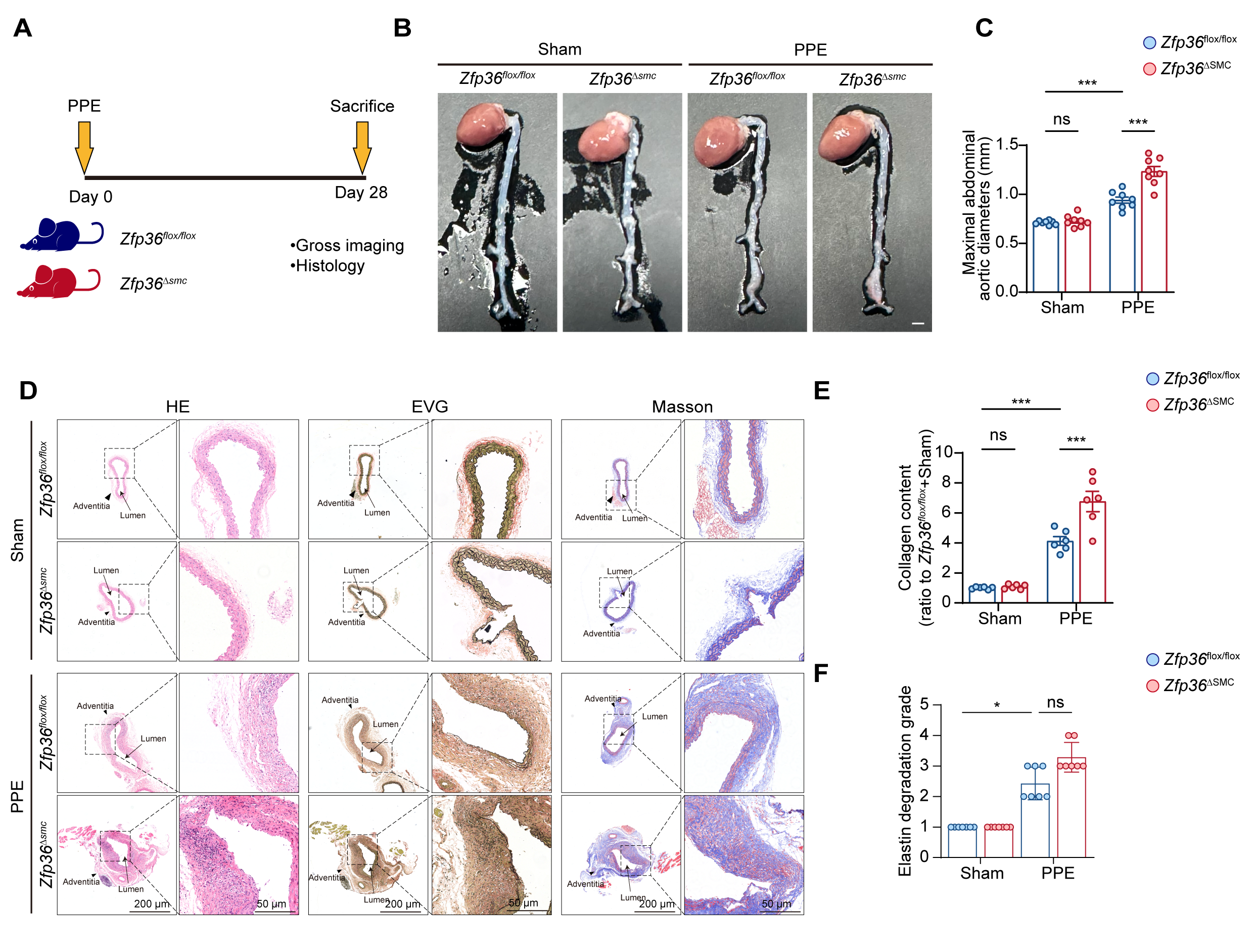


**Figure S2. VSMC-specific *Zfp36* deletion augmented elastase induced AAA.**

**A**, Diagram of experiments procedure. **B**, Representative images of macroscopic features of abdominal aortas. Scale bar indicates 2 mm. **C**, Quantification of the maximal diameter of infrarenal abdominal aortas (n=8 per group). Data was analyzed by two-way analysis of variance (ANOVA) following Tukey’s multiple comparisons. **D**, Representative images of HE, EVG and Masson staining of crossed-sections of abdominal aortas. **E**, Quantitative analysis of collagen deposition (n=6 per group). Data was expressed as the mean ± SEM and analyzed by two-way analysis of variance (ANOVA) following Tukey’s multiple comparisons. **F**, Quantitative analysis of elastin degradation (n=6 per group). Data was analyzed by Nonparametric Kruskal–Wallis test with Dunn’s post-hoc test. Ns indicates no significant; **P* < 0.05; ****P* < 0.001.


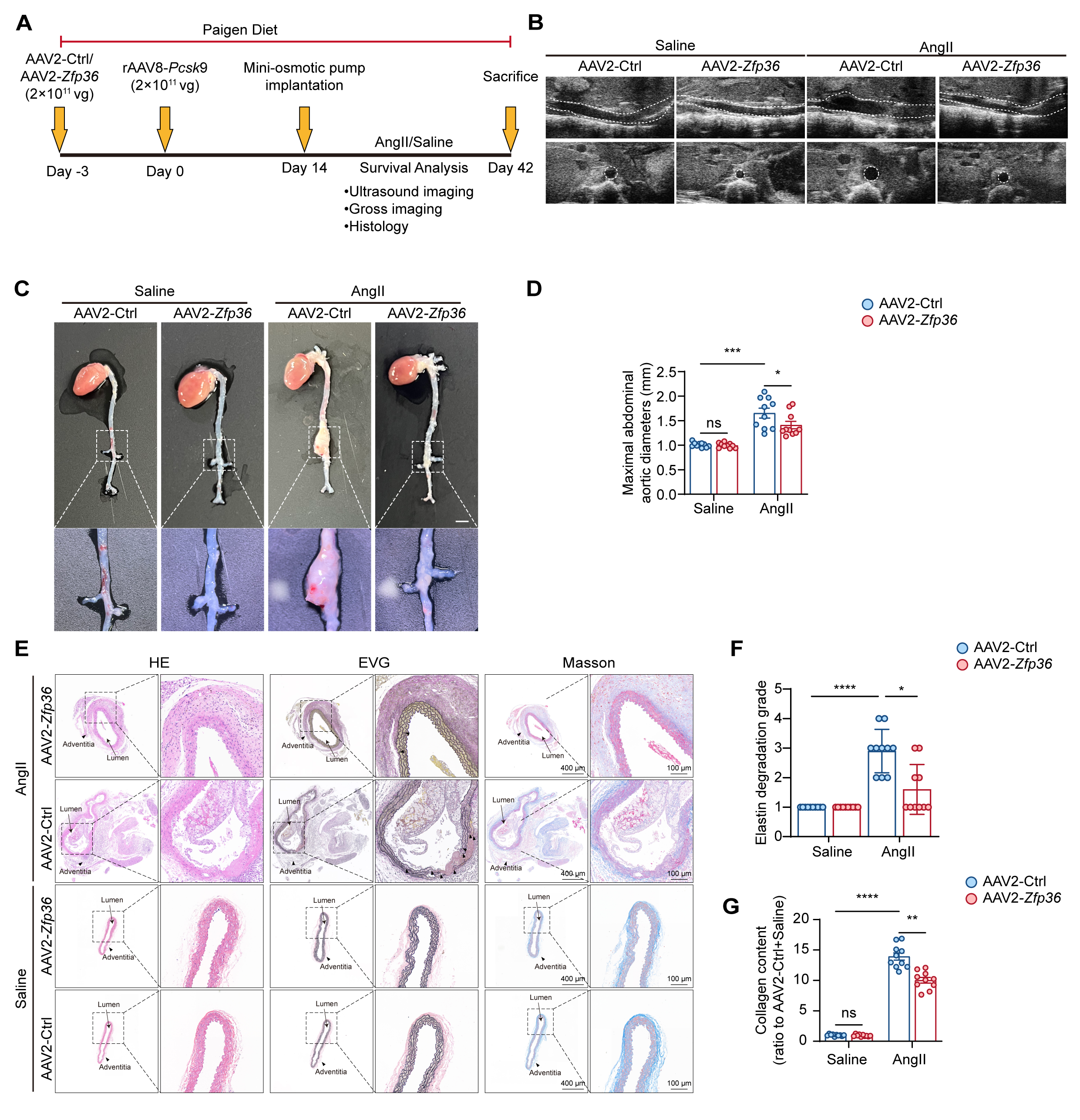


**Figure S3. VSMC-specific *Zfp36* overexpressing inhibits AAA formation.**

**A**, Diagram of experiments procedure. **B**, Representative images of abdominal aortas visualized by using the ultrasound imaging in indicated groups. **C**, Representative images of macroscopic features of abdominal aortas (n=10 per group). Scale bar indicates 2 mm. **D**, Quantification of the maximal diameter of suprarenal abdominal aortas (n=10 per group). Data was analyzed by two-way analysis of variance (ANOVA) following Tukey’s multiple comparisons. **E**, Representative images of HE, Masson and EVG staining of crossed-sections of abdominal aortas. **F**, Quantitative analysis of collagen deposition (n=10 per group). Data was expressed as the mean ± SEM and analyzed by two-way analysis of variance (ANOVA) following Tukey’s multiple comparisons. **G**, Grade of elastin degradation (n=10 per group). Data was analyzed by Nonparametric Kruskal-Wallis test with Dunn’s *post-hoc* test. Ns indicates no significant; **P* < 0.05; ***P* < 0.01; ****P* < 0.001.


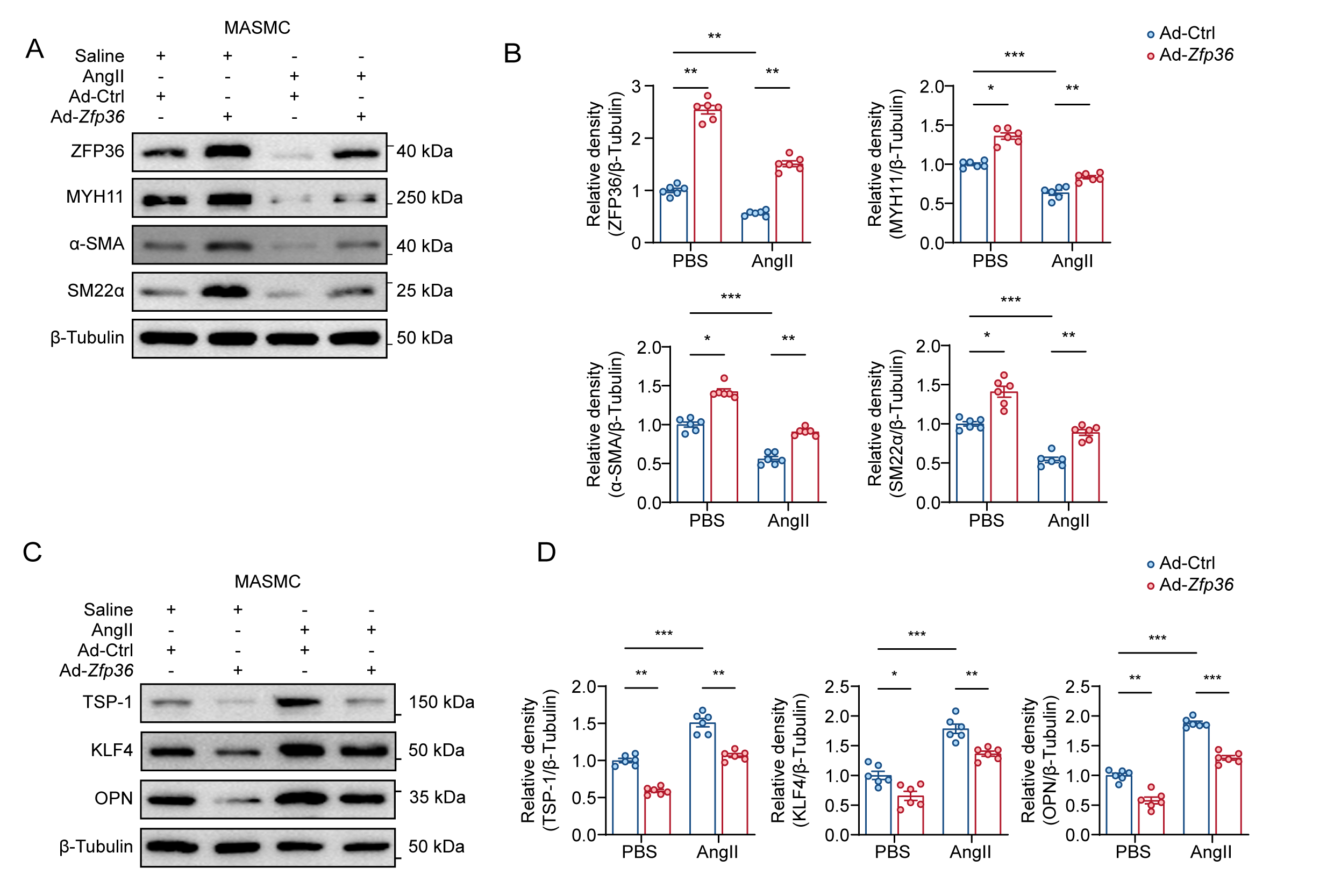


**Figure S4. ZFP36 overexpressing preserved VSMC contractile phenotype.**

**A**-**B**, Expressions of contractile phenotype related proteins of VSMCs infected with Ad-Ctrl or Ad -*Zfp36* and treated with Saline or AngII (1μM) for 48h (n=6 per group). and quantification of protein expression levels. **C**-**D**, Expressions of synthetic phenotype related proteins of VSMCs infected with Ad-Ctrl or Ad-*Zfp36* and treated with Saline or AngII (1μM) for 48h (n=6 per group), and quantification of protein expression levels. Statistical analyses of **B** and **D** were performed by two-way analysis of variance (ANOVA) following Tukey’s multiple comparisons. **P* < 0.05; ***P* < 0.01; ****P* < 0.001.


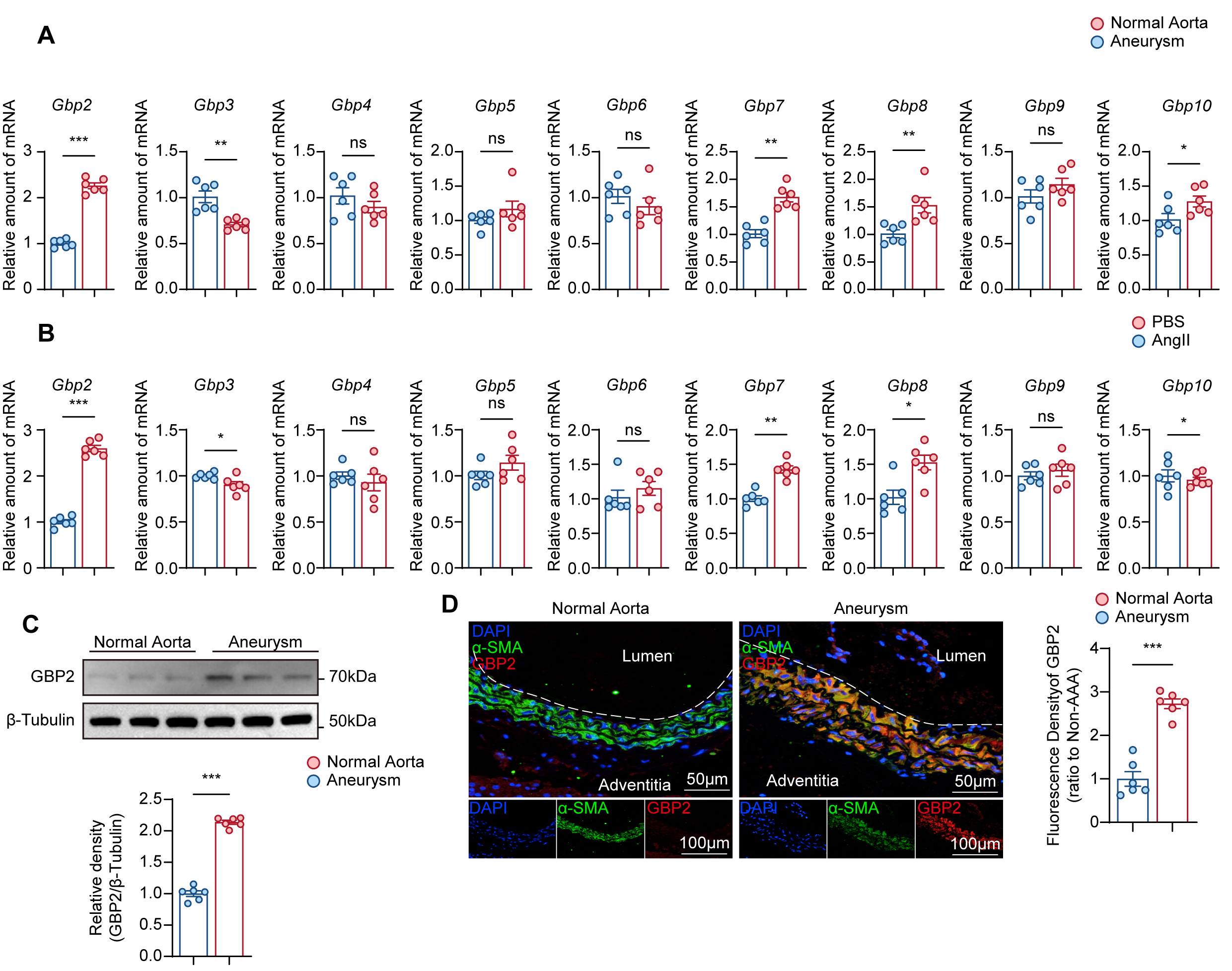


**Figure S5. GBP2 is significantly up-regulated during AAA formation.**

**A**, Relative mRNA levels of *Gbp2-Gbp10* in aortic tissues of Aneurysm or Normal Aorta (n=6 per group). **B**, Relative mRNA levels of *Gbp2-Gbp10* in VSMCs treated with PBS or AngII (1μM) for 48h (n=6 per group). **C**, Expressions of GBP2 in aortic tissues of Aneurysm or Normal Aorta (n=6 per group). **D**, Representative images of immunofluorescence staining of aortic sections of Aneurysm or Normal Aorta and the quantification (n=6 per group). Statistical analyses of **A**, **B**, **C**, and **D** were performed by unpaired *t* test. Ns indicates no significant; **P* < 0.05; ***P* < 0.01; ****P* < 0.001.


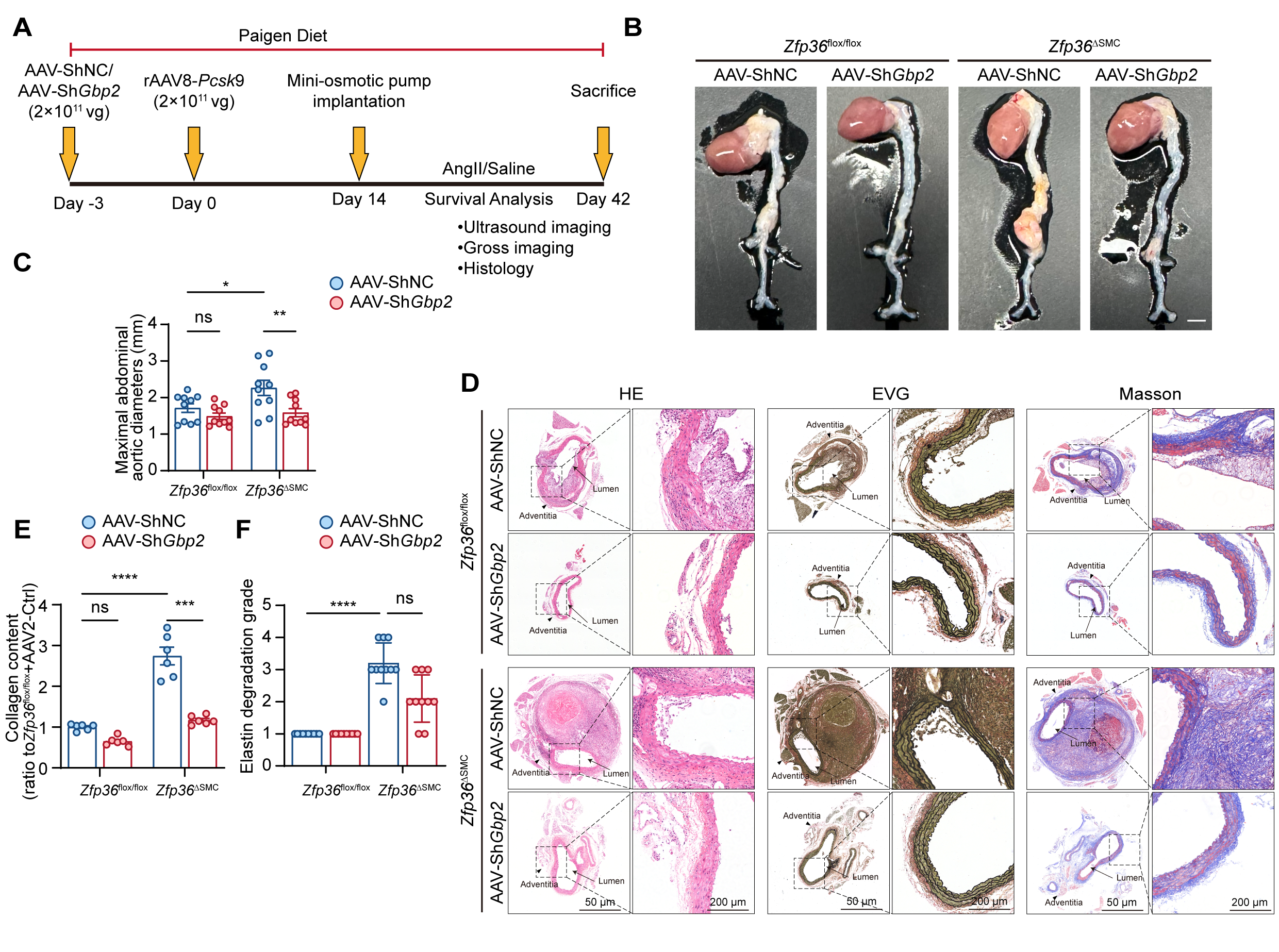


**Figure S6. GBP2 knockdown in VSMCs rescued *Zfp36* deletion induced AAA progression.**

**A**, Diagram of experiments procedure. **B**, Representative images of macroscopic features of abdominal aortas (n=10 per group). Scale bar indicates 2 mm. **C**, Quantification of the maximal diameter of infrarenal abdominal aortas (n=10 per group). Data was analyzed by two-way analysis of variance (ANOVA) following Tukey’s multiple comparisons. **D**, Representative images of HE, EVG and Masson staining of crossed-sections of abdominal aortas. **E**, Quantitative analysis of collagen deposition (n=10 per group). Data was expressed as the mean ± SEM and analyzed by two-way analysis of variance (ANOVA) following Tukey’s multiple comparisons. **F**, Quantitative analysis of elastin degradation (n=10 per group). Data was analyzed by Nonparametric Kruskal-Wallis test with Dunn’s *post-hoc* test. Ns indicates no significant; **P* < 0.05; ***P* < 0.01; ****P* < 0.001; *****P* < 0.0001.


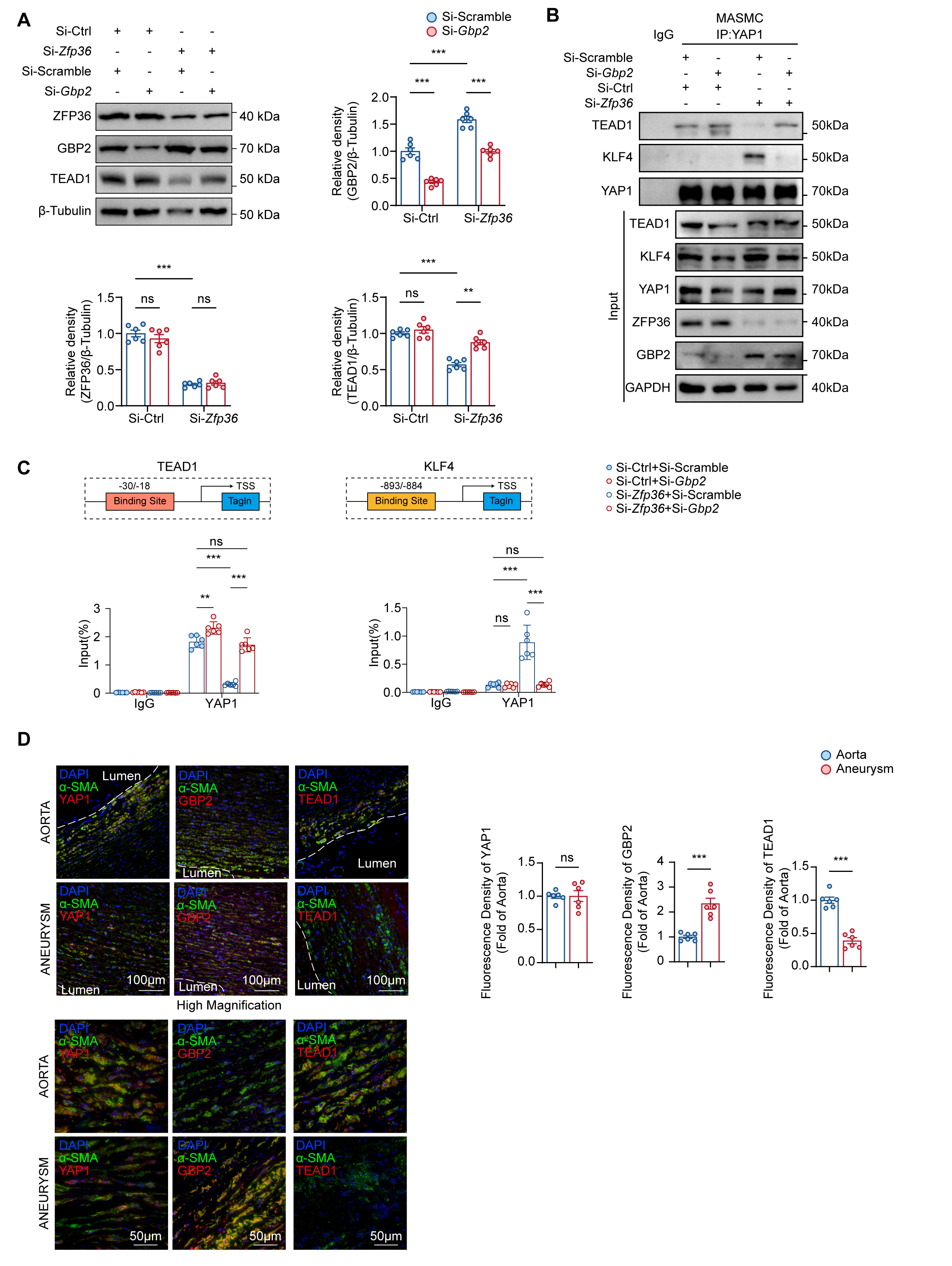


**Figure S7. GBP2 promoted YAP1/KLF4 complex formation.**

**A**, VSMCs were transfected with SI-Scramble, SI-*Zfp36* or SI-*Gbp2* and immunoprecipitated with anti-YAP1 antibody. **B**, Protein levels of VSMCs transfected with Si-*Zfp36* and Si-*Gbp2* (n=6 per group). **C**, ChIP was performed with antibodies to YAP1 or IgG, and the TEAD1 or KLF4 target promoter regions of *Tagln* was amplified by qPCR (n=6 per group). Statistical analyses of **A** and **C** were performed using two-way analysis of variance (ANOVA) following Tukey’s multiple comparisons. **D**, Representative images of YAP1, GBP2, TEAD1 expression by IF staining of human AAA and normal aorta sections and quantification of fluorescence intensity (n=6 per group). Statistical analyses of were performed using unpaired *t* test. Ns indicates no significant; **P* < 0.05; ***P* < 0.01; ****P* < 0.001

.


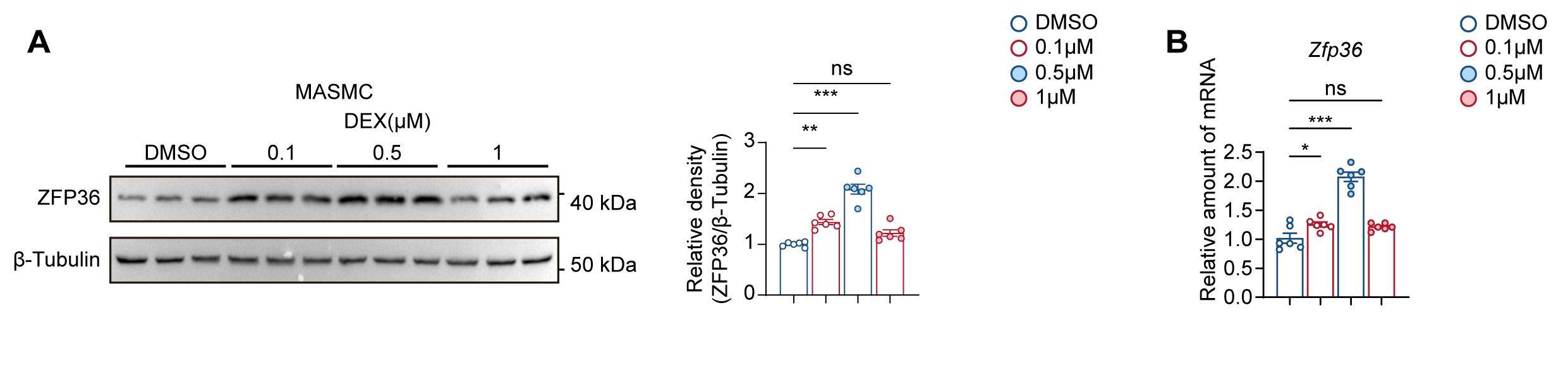


**Figure S8. Dexamethasone promoted NR3C1 expression in VSMCs.**

**A**, Expressions of ZFP36 in VSMCs treated with dexamethasone of different doses for 48h (n=6 per group). **B**, Relative mRNA levels of *Zfp36* in VSMCs treated with dexamethasone of different doses for 48h (n=6 per group). Statistical analyses of **A** and **B** were analyzed by one-way analysis of variance analysis of variance (ANOVA). Ns indicates no significant; **P* < 0.05; ***P* < 0.01; ****P* < 0.001.


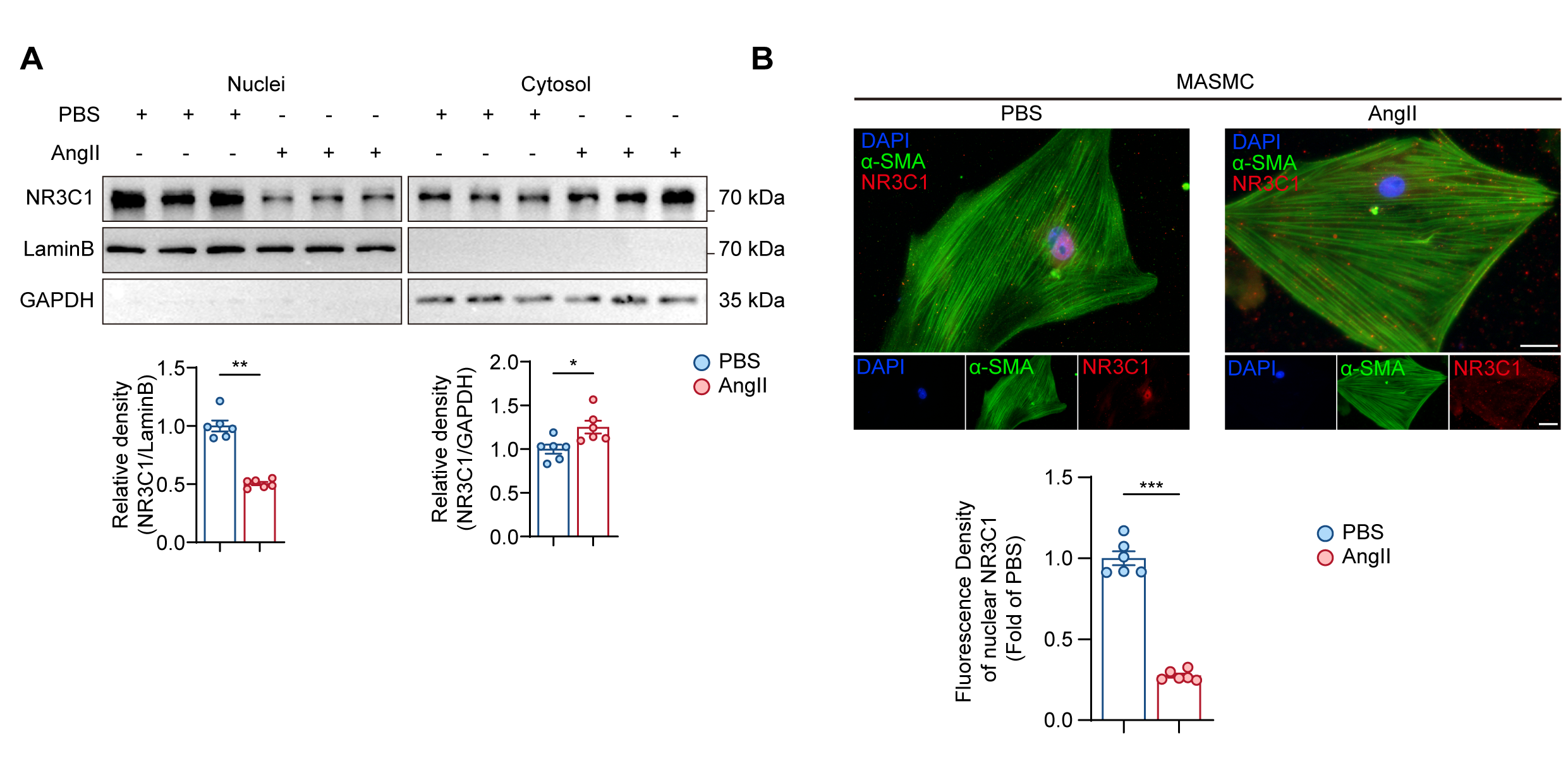


**Figure S9.** **AngII repressed NR3C1 nuclear location and ZFP36 expression in an AT1R dependent manner.**

**A**, Nuclear and cytoplasmic protein levels of VSMCs treated with PBS or AngII (1μM) for 48h (n=6 per group). **B**, Representative images of NR3C1 expression by IF staining of VSMCs treated with PBS or AngII (1μM) for 48h (n=6 per group), co-stained with VSMC marker α-SMA and DAPI. Scale bar indicates 20μm. Quantification of fluorescence intensity of NR3C1 (n=6 per group). Statistical analyses of **A** and **B** were performed by unpaired *t* test. **P* < 0.05; ***P* < 0.01; ****P* < 0.001.


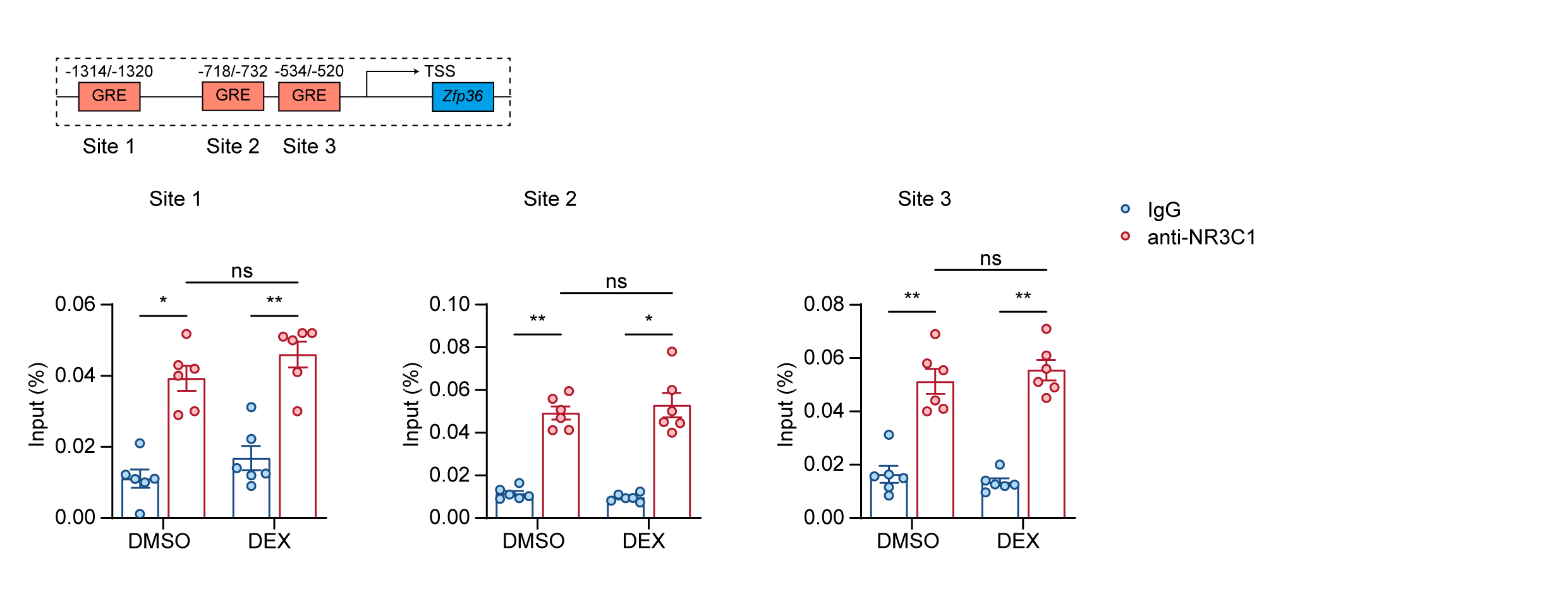


**Figure S10. ChIP-qPCR results of NR3C1 on *Zfp36* promotor.**

The binding between NR3C1 and other three GREs within *Zfp36* promotor was examined by ChIP-qPCR (n=6 per group). Statistical analysis was performed by two-way analysis of variance (ANOVA) following Tukey’s multiple comparisons. Ns indicates no significant; **P* < 0.05; ***P* < 0.01.


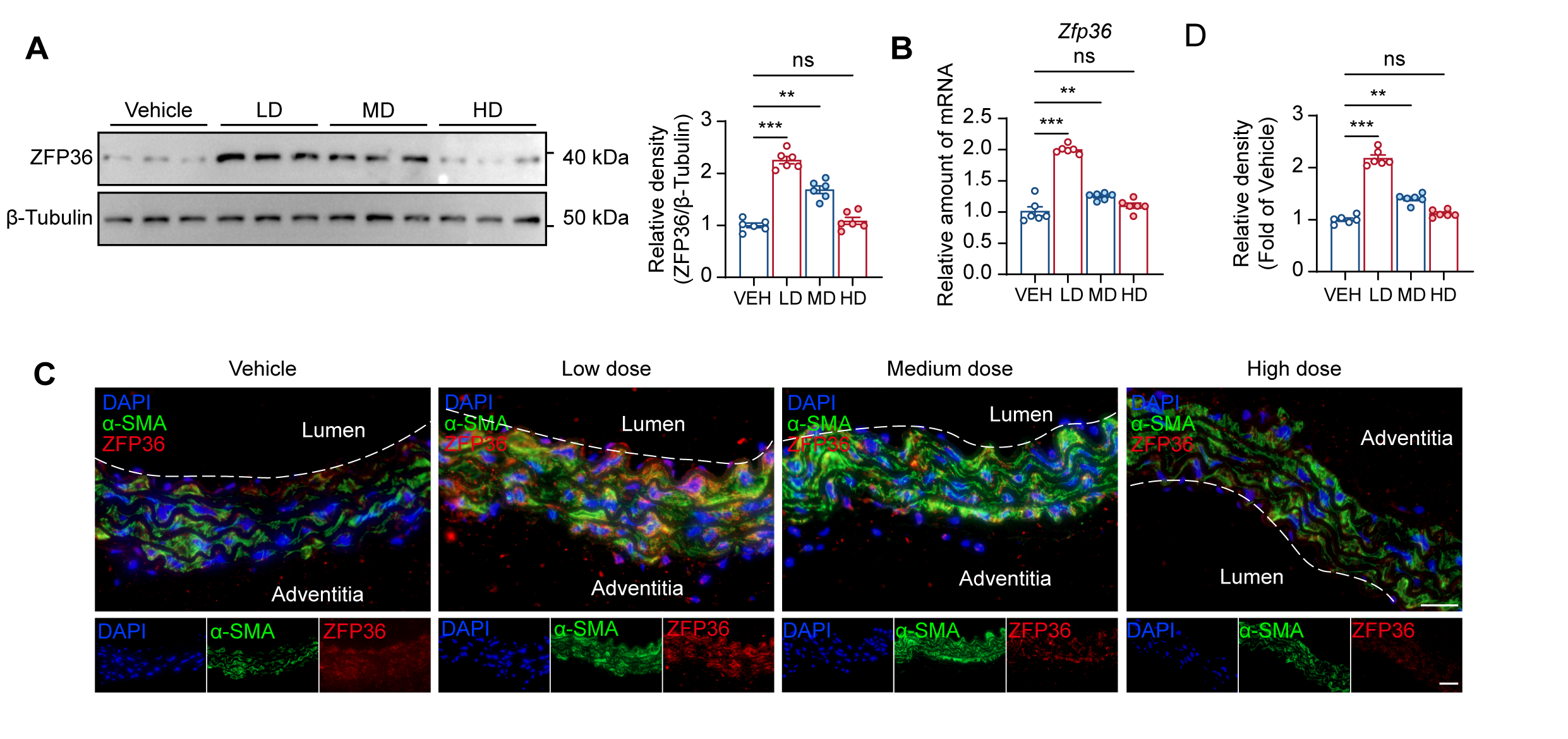


**Figure S11. Administration of low dose dexamethasone promoted ZFP36 expression *in vivo*.**

**A**, Expressions of ZFP36 of aortic tissues from mice treated with saline or dexamethasone of low dose (10 μg/kg/d), medium dose (20 μg/kg/d), high dose (50 μg/kg/d) for 28d (n=6 per group). **B**, Relative mRNA levels of *Zfp36* of aortic tissues from mice treated with dexamethasone of different doses for 28d (n=6 per group). **C-D**, Representative images of immunofluorescence staining of aortic sections from mice treated with dexamethasone of different doses for 28d (n=6 per group) and fluorescence density quantification. Statistical analyses of **A**, **B**, and **D** were performed by a one-way analysis of variance analysis of variance (ANOVA). Ns indicates no significant; ***P* < 0.01; ****P* < 0.001.
